# Biaryl Sulfonamides Based on the 2-Azabicycloalkane Skeleton—Synthesis and Antiproliferative Activity

**DOI:** 10.3390/ma13215010

**Published:** 2020-11-06

**Authors:** Dominika Iwan, Karolina Kamińska, Elżbieta Wojaczyńska, Mateusz Psurski, Joanna Wietrzyk, Marek Daszkiewicz

**Affiliations:** 1 Faculty of Chemistry, Wrocław University of Science and Technology, Wybrzeże Wyspiańskiego 27, 50-370 Wrocław, Poland; dominika.iwan@pwr.edu.pl (D.I.); karolina.kaminska@pwr.edu.pl (K.K.); 2 Department of Experimental Oncology, Hirszfeld Institute of Immunology and Experimental Therapy, Polish Academy of Sciences, Rudolfa Weigla 12, 53-114 Wrocław, Poland; mateusz.psurski@hirszfeld.pl (M.P.); joanna.wietrzyk@hirszfeld.pl (J.W.); 3 Institute of Low Temperature and Structure Research, Polish Academy of Sciences, Okólna St. 2, 50-422 Wrocław, Poland; marek.daszkiewicz@intibs.pl

**Keywords:** antiproliferative activity, azanorbornane derivatives, bicyclic compounds, sulfonamide

## Abstract

In a search for new, selective antitumor agents, we prepared a series of sulfonamides built on bicyclic scaffolds of 2-azabicyclo(2.2.1)heptane and 2-azabicyclo(3.2.1)octane. To this end, *aza*-Diels–Alder cycloadducts were converted into amines bearing 2-azanorbornane or a bridged azepane skeleton; their treatment with sulfonyl chlorides containing biaryl moieties led to the title compounds. The study of antiproliferative activity of the new agents showed that some of them inhibited the growth of chosen cell lines with the IC_50_ values comparable with cisplatin, and some derivatives were found considerably less toxic for nonmalignant cells.

## 1. Introduction

The development of pharmaceuticals used for cancer treatment is connected with an introduction of various classes of antineoplastic drugs: metal complexes, alkylating agents, antimetabolites, natural products, etc. The success of certain chemotherapeutics like cisplatin is undoubtful, but side effects and drug resistance problems prompt chemists to search for new alternatives with comparable activity against malignant cells, but increased selectivity [1,2]. Sulfonamides, generally associated with their traditional use as antibacterial drugs, were found to exhibit a multidirectional biological activity [3,4], including the ability to inhibit the growth of selected tumors [5,6,7,8,9]. Besides the main functional group present in the molecule, also certain structural motives were identified as beneficial for the desired therapeutic action. Biphenyl, or, more general, biaryl moiety is found in many natural and synthetic compounds of biomedical interest. As an example, Luo and co-workers identified two new ATP-competitive inhibitors of kinesin spindle protein which plays an essential role in the early stages of mitosis [10]. These compounds containing a biphenyl fragment appeared efficient against colorectal cell line. Based on this discovery, urea and thiourea derivatives bearing this substituent were tested in vitro by Holland, Fischer, and co-workers using prostate, ovarian, and breast cancer cells, with three compounds showing desired activity and selectivity [11]. In another study by Zhang and co-workers, biphenyl-based ureas displayed potent ability to inhibit the proliferation of human lung cancer cells A549 and human hepatoma cells [12]. One of biphenyl methylene indolones tested by Donthiboina et al. was found a particularly efficient growth inhibitor of HeLa and prostate cell line DU-145 [13].

In our research, we focus on a preparation and applications of multifunctionalized compounds based on a chiral, bicyclic skeleton. 2-azabicyclo(2.2.1)heptane (2-azanorbornane), synthetically available through a stereoselective aza-Diels–Alder reaction, is regarded as a versatile platform for the synthesis of a variety of derivatives for the use in asymmetric synthesis, but also biomedical studies [14]. Importantly, due to the multiplication of chirality in the cycloaddition step, a single enantiomer bearing four stereogenic centers with a defined configuration can be isolated [14,15,16,17,18,19]. The skeleton of the base compound can be appropriately modified either by introduction of various groups, typically into position 3 or 2, formation of dimeric species, and extending of the bicyclic system (in a stereoselective manner) to 2-azabicyclo(3.2.1)octane [20]. Certain functionalities, like hydroxyl or amine groups, offer a convenient access to a set of other derivatives; on the other hand, the presence of the amine moiety opens the possibility of interactions (e.g., by the formation of hydrogen bonds) in a biological system.

In our previous study on the biomedical applications of 2-azabicycloalkane derivatives a series of sulfonamides based on the bicyclic chiral scaffold were prepared [21]. The cytotoxicity of the synthesized, stable products was tested using human cancer cell lines: glioblastoma (GBM) medulloblastoma (MB), and hepatocellular carcinoma (HCC), and several sulfonamides were found to exhibit significant cytotoxicity. However, as we concluded, discrimination of toxicity between malignant and nonmalignant cells was too narrow (less than >30-fold difference which is suggested on security grounds [22]). Thus, we decided to introduce appropriate modifications to the structures of the sulfonamides, including substituents of SO_2_ fragment, but also at N-2 of the bicyclic system, and the linker between the skeleton and a functional group. The results of our exploration are presented in this contribution.

## 2. Materials and Methods

### 2.1. General Considerations

All the solvents and reagents were received from chemical companies and we used them without additional purification. Schmelzpunkt Bestimmer Apotec melting point apparatus (WEPA Apothekenbedarf GmbH & Co. KG., Hillscheid, Germany) was used for the determination of melting points in a standard open capillary. ^1^H and ^13^C NMR spectra were recorded on Jeol 400yh (Jeol Ltd., Tokyo, Japan), Bruker Avance III500, and Bruker Avance II 600 spetrometers (Bruker, Billerica, MA, USA). The residual ^1^H or ^13^C signals of the solvent (chloroform-*d*) were used as references. Chemical shifts are given in ppm, and coupling constants are expressed in Hz. The spectra are shown in Figures S1–S18. High-resolution mass spectra were recorded using electrospray ionization mode on a Waters LCT Premier XE TOF spectrometer (Waters Corporation, Milford, MA, USA). Infrared spectra were measured in a 4000–400 cm^−1^ range on a Perkin Elmer 2000 FTIR instrument (PerkinElmer, Waltham, MA, USA). Optical rotations were determined with an automatic polarimeter Model AA-5 (Optical Activity, Ltd., Ramsey, UK); [α]_D_ values are expressed in 10^−1^ deg cm^2^ g^−1^. Silica gel 60 (60–200 µm, 70–230 mesh) was used for column chromatography, and precoated plates precoated with the same adsorbent were apllied for thin-layer chromatography.

Four-circle single crystal diffractometer (Oxford Diffraction Ltd., Wrocław, Poland) with a CCD Atlas detector using graphite-monochromatized Mo*K*_α_ radiation (λ = 0.71073 Å) was applied for the collection of X-ray diffraction data. The raw data were processed with the CrysAlis Data Reduction Program (version 1.171.39.46). Corrections for polarization and Lorentz effects were introduced to reflection intensities. The crystal structure was solved by direct methods with SHELXS-2018/3 and refined using full-matrix least-squares method using SHELXL-2018/3 program [23]. Anisotropic displacement parameters were applied for the refinement of non-hydrogen atoms. H-atoms, though visible on the Fourier difference maps, were placed by geometry and allowed refined ‘riding on’ the parent atom with U_iso_ = 1.2 U_eq_(C) for CH and CH_2_ groups and U_iso_ = 1.5 U_eq_(C) for CH_3_ groups. Coordinates of hydrogen atom of the N–H group was refined for **9i**, but U_iso_ = 1.2 U_eq_(N). In the case of compound **10i**, hydrogen atom position of the N–H groups were fixed at values found for maximum at the difference map and U_iso_ = 1.5 U_eq_(N). The reflection intensities were treated by the PLATON program (version 281019) with ‘squeeze’ procedure, because the position of a solvent molecule was not determined. The details of crystal data and structure refinement are presented in Table S1. Structures were visualized using Diamond 3.2k [24].

### 2.2. Preparation of Starting Compounds

Synthesis of bicyclic amines, (1*S*,4*S*,5*R*)-2-[(*S*)-1-phenylethyl]-4-amine-2-azabicyclo(3.2.1)octane **4**, and (1*S*,3*R*,4*R*)-2-[(*S*)-1-phenylethyl]-3-aminemethyl-2-azabicyclo(2.2.1)heptane **6** was reported in our previous papers [20,25,26]. Preparation of enantiomerically pure 2-azanorbornane amine derivatives with two bicyclic subunits was described as well [25]. Amine **8** was prepared from (1*S*,4*S*,5*R*)-4-chloro-2-((S)-1-phenylethyl)-2-azabicyclo(3.2.1)octane [20] via the reaction with potassium cyanide followed by reduction with LiAlH_4_ (yield 64%). Amine **5** was obtained using an adapted procedure from the patent [27].

### 2.3. General Procedure for Sulfonamide Synthesis

Primary amine (1.0 mmol) and KOH (powdered, 0.10 g, 1.8 mmol, 1.8 equiv.) were dissolved in dry CH_2_Cl_2_ (15.0 mL). A chosen sulfonyl chloride (1.0 mmol, 1.0 equiv.) was then introduced. The mixture was stirred for 24 h at room temperature. After addition of brine the mixture was extracted with dichloromethane. The organic phases were dried (Na_2_SO_4_), and evaporated to dryness after filtration through Celite^®^. Thus obtained crude sulfonamides were purified using column chromatography on silica, and eluted with ethyl acetate/*n*-hexane (1:1 *v/v*).

The details of experimental procedures and the relevant physicochemical data of all newly prepared compounds together with copies of ^1^H and ^13^C NMR spectra are gathered in Supplementary Materials.

### 2.4. Biological Activity Analysis

#### 2.4.1. Cell Culture

The following human cancer cell lines, representing the most common types of cancer, were chosen for evaluation of the antiproliferative activity of the prepared sulfonamides: lung cancer (A549), breast cancer (MCF-7), colon adenocarcinoma (LoVo), and biphenotypic B cell myelomonocytic leukemia (MV4-11). As a reference, the normal mouse fibroblasts cell line (BALB/3T3) was applied. A549, LoVo and BALB/3T3 cell lines were purchased from the ATCC (American Type Culture Collection, Rockville, MD, USA), MCF-7 cell line—from EACC (The European Collection of Cell Cultures), and MV4-11 cell line was obtained from DSMZ (Leibniz Institute—German Collection of Microorganisms and Cell Culture, Braunschweig, Germany). All these lines were maintained at the Hirszfeld Institute of Immunology and Experimental Therapy (IIET) in Wrocław, Poland.

We used the following culture media: for A549 cell line: the mixture of RPMI 1640 and OptiMEM (1:1) medium (both from Gibco, Scotland, UK)), 2mM l-glutamine, 1mM sodium pyruvate (Sigma-Aldrich Chemie GmbH, Steinheim, Germany) and 5% fetal bovine serum (FBS; HyClone Laboratories, Logan, UT, USA). MCF-7 cells were cultured in Eagle’s medium (IIET, PAS, Wroclaw, Poland), 2mM l-glutamine and 10% of FBS supplemented with 0.8 mg/L of insulin (Sigma-Aldrich Chemie GmbH, Steinheim, Germany). For LoVo cell line, F12K medium was applied (American Type Culture Collection, Rockville, MD, USA-ATCC), with 5% FBS (HyClone Laboratories, Logan, UT, USA). MV4-11 cells were cultured in RPMI 1640/GlutaMax I medium (Gibco, Thermo Fisher Scientific, Leicestershire, UK) with 1 mM sodium pyruvate (Sigma-Aldrich Chemie GmbH, Steinheim, Germany) and 10% FBS (HyClone Laboratories). In case of BALB/3T3 cells, DMEM (Gibco, Thermo Fisher Scientific) supplemented with 2 mM l-glutamine and 5% FBS was used. Antibiotics: 100 U/mL penicillin (Polfa Tarchomin SA, Warsaw, Poland) and 100 µg/mL streptomycin (Sigma-Aldrich Chemie GmbH, Steinheim, Germany) were present in all the culture media; cells were cultured in a humid atmosphere containing 5% CO_2_ at 37 °C.

#### 2.4.2. In Vitro Anti-Proliferative Assays 

Twenty-four hours before addition of the tested compounds, each cell line was seeded in 96-well plastic plates (Sarstedt, Numbrecht, Germany) in an appropriate medium at 10^4^ cells/well density, except MCF7 cell line: 0.75 × 10^4^/well, and A549 cell line: 0.25 × 10^4^/well. The studied cell lines were subjected to each of the tested sulfonamides at 4 different concentrations in the range of 100–0.1 µg/mL for 72 h. Cisplatin (Teva Pharmaceuticals, Poland) was used as a reference compound, and DMSO (Sigma-Aldrich Chemie GmbH, Steinheim, Germany) served as a solvent control at concentrations equivalent to these applied in the solutions of the tested sulfonamides. MTT assay was performed for leukemic cells, while sulforhodamine B assay (SRB) for the adherent cells.

#### 2.4.3. SRB Assay

After 72 h of incubation, cells were fixed in situ by gently adding of 50 µL per well of ice-cold 50% TCA (trichloroacetic acid, POCh, Gliwice, Poland) and were incubated at 4 °C for one hour. Each well was then washed five times with water, followed by addition of 50 µL of 0.4% solution of SRB (sulforhodamine B, Sigma-Aldrich Chemie GmbH, Steinheim, Germany) in 1% acetic acid (POCh, Gliwice, Poland), and plates were again incubated at RT for 0.5 h. Plates were washed five times with 1% acetic acid to remove the unbound dye. The stained cells were treated with 10 mM TRIS (Tris base, Sigma-Aldrich, Chemie GmbH, Steinheim, Germany). Absorbance at 540 nm in each well was read with Elisa plate reader (BioTek Synergy H4, Swindon, UK) using the Gen5 software [28].

#### 2.4.4. MTT Assay

To each well, 20 µL of 3-(4,5-dimethylthiazol-2-yl)-2,5-diphenyl tetrazolium bromide solution (Sigma-Aldrich, Chemie GmbH, Steinheim, Germany) was added. Plates were incubated for 4 h at 37 °C, and then centrifuged for 5 min, at 88× g, at 4 °C. The supernatant was discarded, and 200 µL of DMSO per well (POCh, Gliwice, Poland) was added. After 10 min at RT, absorbance at 570 nm was read using the plate reader (BioTek Synergy H4, Swindon, UK), uning Gen5 software [29].

The results are shown as average IC_50_ values (concentration of the compound causing inhibition of cell proliferation by 50%) ± standard deviation. IC_50_ values were determined using the Prolab-3 system based on Cheburator 0.4, a software developed by Nevozhay [30]. At each concentration, sulfonamides were tested in triplicates in a single experiment. Three independent repetitions were applied for each experiment.

## 3. Results and Discussion

### 3.1. Preparation of Compounds

**Scheme 1:**
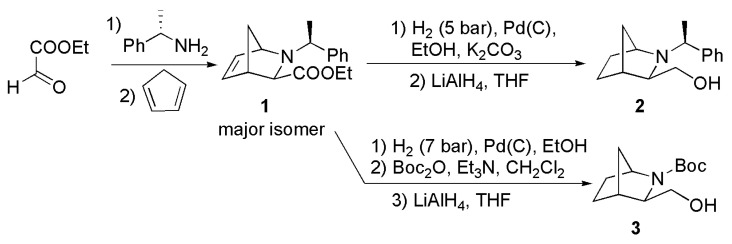
Synthesis of alcohols **2** and **3**.

**Scheme 2:**
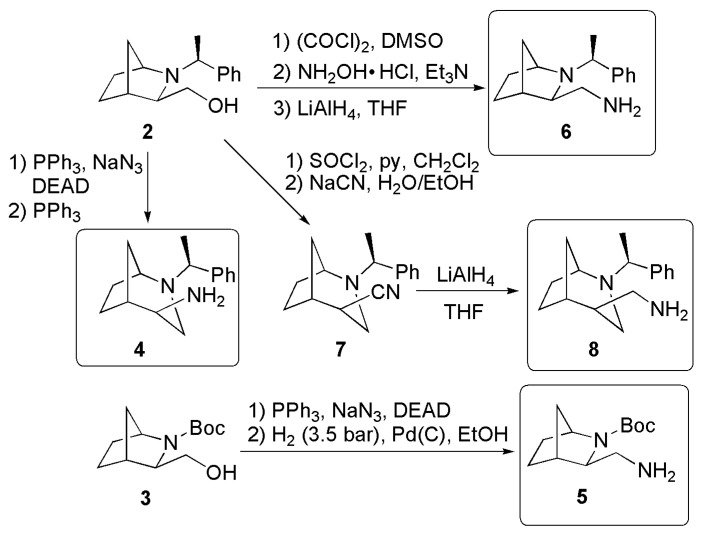
Preparation of amines **4**, **5**, **6**, and **8**.

Enantiomerically pure sulfonamides based on two bicyclic scaffolds, 2-azabicyclo(2.2.1)heptane and 2-azabicyclo(3.2.1)octane, were prepared by an appropriate modification of aza-Diels–Alder cycloadducts as described in our previous publication [21]. The protocol started from a stereoselective reaction between cyclopenatdiene and an enantiopure imine (prepared in situ from (*S*)-1-phenylethylamine and ethyl glyoxylate); the major product, exo isomer **1**, was isolated and used in further transformations (Scheme 1) [14,15,16,17,18,19]. Its reduction, depending on conditions applied, led either to alcohol **2** with a preserved N-substituent, or to product **3** in which the group was removed and replaced with Boc protection (Scheme 1). In the case of derivative **2** its reaction with azide under Mitsunobu conditions resulted in ring expansion, yielding—after reduction—a bridged azepane bearing amine function **4** [20]. The reaction was shown to proceed through aziridinium intermediate formed by the attack of N-2 nucleophile on a carbon atom of the substituent in position 3. In contrast, compound **3** underwent a smooth nucleophilic substitution without alteration of the bicyclic system (Scheme 2). This effect can be attributed to the electron withdrawing properties of tert-butoxycarbonyl group which decreases the nucleophilic character of N-2 center. Thus obtained azide was readily converted to the corresponing amine **5**. Its isomer with a 2-azabicyclo(2.2.1.)heptane scaffold **6** was prepared as described in our prevoious works: Swern oxidation followed by transformation of the formed aldehyde to oxime and its reaction with LiAlH_4_ [27]. Finally, a homologue of amine **4** with an additional methylene group in the substituent was obtained from alcohol **2** in three steps: reacton with sulfonyl chloride in the presence of pyridine resulted in ring expansion and introduction of Cl substituent which was then replaced with cyanide to afford nitrile **7**. Its reduction provided amine **8** which combined structural features of two isomeric compounds **4** and **6**: an enlarged ring and amino group attached to it through a flexible spacer.

**Scheme 3:**
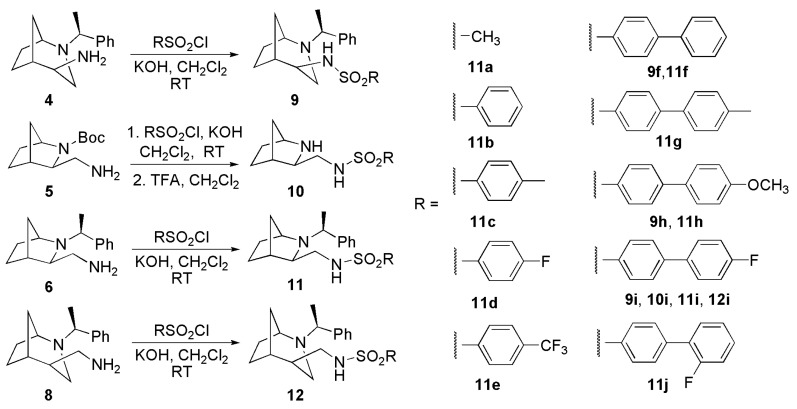
Preparation of sulfonamides **9**–**12**.

Having in hand four amines (**4**, **5**, **6**, and **8**), we reacted them with chosen sulfonyl chlorides, affording coresponding sulfonamides in 70–80% yield in most cases (Scheme 3). The choice of reagents used was based on the results of our previous investigations as well as literature precedents. We expected that introducing biaryl substituents, and, in particular, fluorinated biaryls, should result in an enhanced antiproliferative activity.

All new compounds were fully characterized using spectroscopic methods (IR, ^1^H and ^13^C NMR, HRMS). In addition, for two fluorinated derivatves, **9i** and **10i**, we obtained crystals suitable for X-ray diffraction measurements; their structures further confirmed the formation of the expected products bearing four (**9i**) or three stereogenic centers (**10i**) of defined configuration: (1*S*,4*S*,5*R*,1’*S*) for **9i** and (1*S*,3*R*,4*R*) in the case of **10i**.

**Figure 1:**
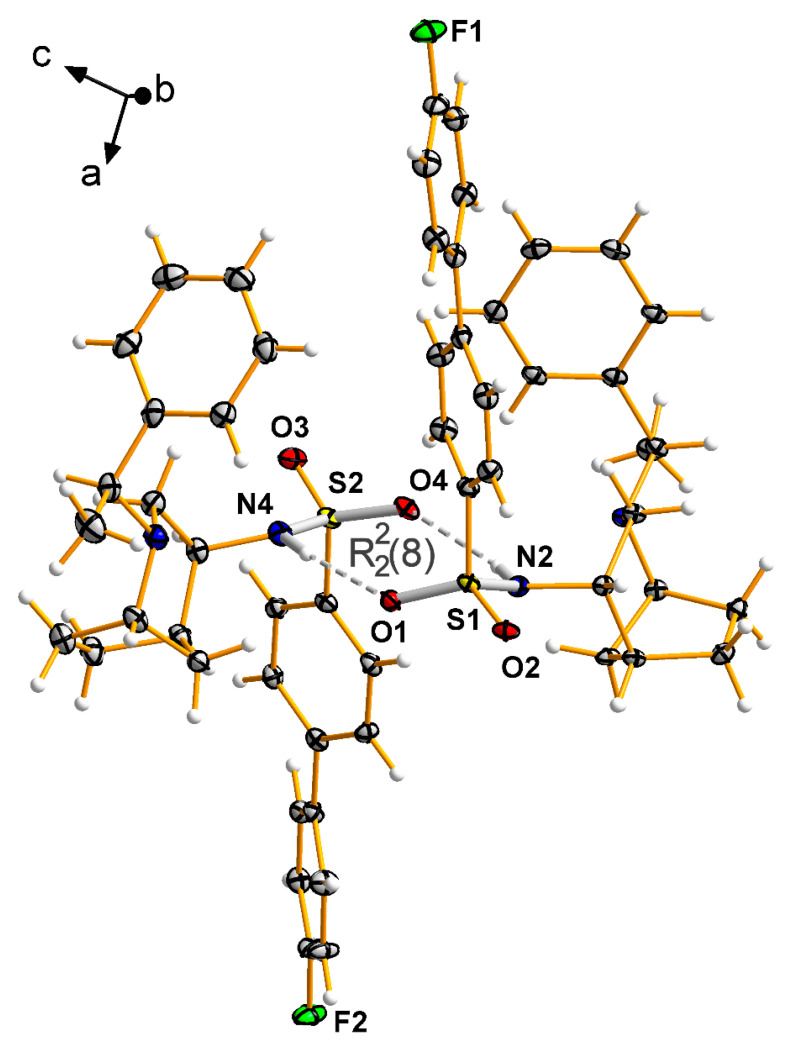
Molecular structure of **9i.** Displacement ellipsoids for non-H atoms drawn at 30% probability level.

**Figure 2:**
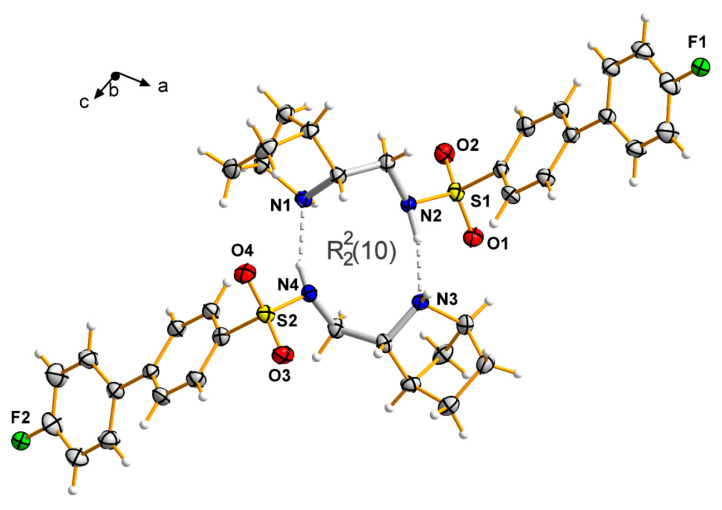
Molecular structure of **10i**. Displacement ellipsoids for non-H atoms drawn at 30% probability level.

The compounds **9i** and **10i** crystallize in enantiomorphic *P*2_1_ and *I*2 space groups, respectively. The crystal structures are non-centrosymmetric, because they consist of only one enantiomer. Selected bond lengths and angles are presented in Table S2. The values are normal and show a well established atomic framework. Geometry parameters for hydrogen bonds indicate that weak intermolecular interactions exist between adjacent molecules (Table S3). The most relevant base on the N–H group, which forms N–H···O hydrogen bond with oxygen atom of the SO_2_ group in **9i**. In the case of **10i**, the nonsubstituted nitrogen atom of the 2-azanorbornane skeleton is an acceptor in N–H···N hydrogen bond. These two types of interactions appear to be of high importance in a view of molecular self-assembly, as they connect two symmetry independent molecules in the crystal structure of **9i** and **10i** and arrange the molecule in dimers (Figure 1 and Figure 2). In the crystal structure of **10i**, the dimers are additionally connected to each other by the N–H···O hydrogen bonds, which stabilize the crystal structure along *b* crystallographic direction. In dimers of **9i** and **10i**, two hydrogen bonds form ring patterns R^2^_2_(8) and R^2^_2_(10), which occur around the non-crystallographic inversion center [31,32].

### 3.2. Antiproliferative Activity of Tested Compounds

Antiproliferative activity of enantiomerically pure sulfonamides **9**–**12** was investigated using human acute myeloid leukemia cell line (MV4-11) and three solid tumor cell lines: lung (A549), colorectal (LoVo), and breast (MCF-7). Normal murine fibroblasts cell line (BALB/3T3) was used for the evaluation of selectivity of the inhibitors. The results are collected in Table 1.

Analysis of the results obtained for a full series of sulfonamides based on one scaffold of 2-azanorbornane (**11a**–**j**) provided a valuable insight into an impact of substitution pattern on the observed antiproliferative action. Among the tested sulfonamides, derivatives **11a**–**c** bearing methyl, phenyl, and tolyl substituents were found practically inactive in concentrations up to 100 µM or exhibited only moderate activity against leukemia cell line (**11b**,**11c**). Introduction of fluorine substituent did not lead to a considerable improvement (compound **11d**). However, trifluoromethyl derivative **11e** was able to inhibit proliferation of all cell lines, with the most promising result for MV4-11 (16.15 ± 4.95 µM). Still, the results for this sulfonamide were significantly surpassed by biaryl-substituted compounds, in agreement with our expectations. All five derivatives **11f**–**j** showed a desired activity, in some cases with IC_50_ value comparable with cisplatin. In particular, the results obtained for compounds **11f** (3.09 ± 1.56 µM) and **11g** (4.26 ± 3.97 µM) are amongst the best in the whole series. On the other hand, the same sulfonamides were much worse in comparison with **11h**–**11j** in the growth inhibition of breast cancer MCF-7. Fluorinated derivative, **11i**, as expected, exhibited a reasonable antiproliferative activity and all IC_50_ values were smaller than 15 µM; the best value 2.98 ± 1.38 µM for A549 line was the lowest among all sulfonamides tested. One should not forget, however, that in many cases a proven performance against cancer cells is accompanied with high toxicity for normal cells (vide infra).

Comparison of compounds bearing the same fluorinated biphenyl substituent, namely **9i**, **10i**, **11i**, and **12i** revealed the influence of the chiral bicyclic scaffold on the utility of these azabicyclic compounds as the possible drug candidates. As it can be seen in Table 1, ring size and flexibility of the connection does not change much the effect of application of given sulfonamide for all tested lines. All fluorinated derivatives exhibited a significant activity comparable with cisplatin, and even in some cases the IC_50_ values were lower (derivative **11i**, A548 and LoVo lines). The presence of N-2 (1-phenylethyl) substituent was generally beneficial since the activity of **10i**, capable of formation of additional hydrogen bonds (as exemplified by its X-ray structure) was decreased in comparison to **11i**, with an exception of MV4-11 leukemia cells. The results for isomers (**9i** vs. **11i**) and homologs (**12i** vs. **11i**) suggested that a bigger bicyclic system with amino group attached directly to it is slightly more active (meaningful differences were found for A549 and LoVo lines).

Finally, we can analyze the effect of substitution of the biaryl system (compounds **9f**, **9h**, and **9i** and **11f**–**11j**). While for the 2-azabicyclo(3.2.1.)octane system the presence of methoxy group resulted in a slight decrease of IC_50_ values (**9f** vs. **9h**), for 2-azanorbornane derivatives the impact of –OCH_3_ fragment varied with the cell line (**10f** vs. **10h**). Similarly, methyl substituent decreases the activity for A549 cells, but increases for LoVo and MCF-7 (**10f** vs. **10g**). Fluorine in biphenyl system of **11** was found beneficial for the treatment of solid tumors (but not for leukemia cells), and placement the halogen in para position seemed better in that case (**11i** vs. **11j**).

**Figure 3:**
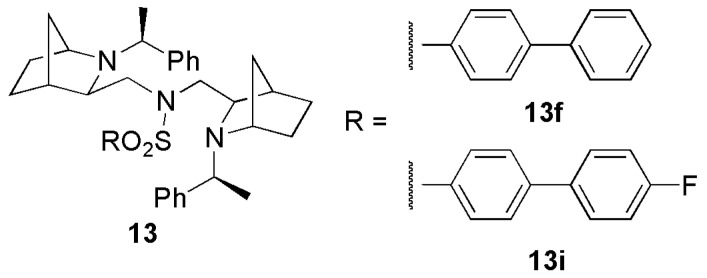
Dimeric derivatives **13f**, **13i**.

Additionally, the results for dimeric species **13f** and **13i**, bearing biphenyl and fluorobiphenyl substituent, respectively (Figure 3) were, to our surprise, practically inactive against the tested cell lines, with **13i** leading to even worse results.

Several conclusions on key structural features can be drawn from such a preliminary SAR (structure–activity relationship) analysis. Biaryl substituent on sulfonamide and *N*-(1-phenylethyl) group can be regarded as prerequisites for the desired activity. To discuss further the effect of introduction of fluorine, we should take into account the selectivity of the tested compounds. Fluorinated derivatives **9i**, **10i**, **11i**, and **12i** are active, but also inhibit proliferation of mouse fibroblasts (treated as an example of nonmalignant cells) to a comparable degree. This renders possible application of these compounds, though, as can be seen from Table 1, cisplatin is also quite toxic for the normal cells. Still, for some of the tested sulfonamides, the selectivity is quite promising: **9h** and **10i** have the IC_50_ value for BALB/3T3 line almost 3 and 4 times bigger, respectively, than for leukemia cells MV4-11. Even better selectivity was observed for 2-azanorbornyl derivatives **11f** and **11g**, much less toxic for mouse fibroblasts (>10 times bigger value of IC_50_ as compared to MV4-11 line, but also noticeably higher than for A549 and LoVo lines). These two biaryl-substituted compounds can be regarded as the bet candidates for the further study. In particular, one factor which was not present in the current study could be checked: the impact of configuration of the four stereocenters on the activity of the investigated sulfonamides. Both replacing (*S*)-ethylphenylamine with its optical antipode (which should result in reversing the configuration of three chiral carbon atoms) and utilizing the minor product (endo isomer) of aza-Diels–Alder reaction in further steps (changing the configuration at C-4) would result in a stereoisomer of given compound with a modified interaction with the receptors of the cancer cells.

## 4. Conclusions

In this study, we focused on the antiproliferative activity of sulfonamides bearing characteristic structural features: a chiral, rigid bicyclic skeleton, and biaryl substituents connected to SO_2_ moiety. Part of the compounds exhibited not only a significant activity against cancer cells, but also were meaningfully less toxic for mouse fibroblasts. Such sulfonamides—in particular, **11f** and **11g** 2-azanorbornyl dervatives—can be regarded as the promising starting point for further modifications.

## Figures and Tables

**Table 1:** Antiproliferative activity of sulfonamides.

Compound	IC_50_ [µM] ± SD [*n* = 5]				
	MV4-11	A549	LoVo	MCF-7	Balb/3T3
**9f**	8.76 ± 3.45	13.63 ± 1.10	14.50 ± 2.54	18.82 ± 3.25	14.09 ± 2.63
**9h**	3.92 ± 1.74	9.13 ± 1.05	11.77 ± 2.46	16.34 ± 2.11	15.09 ± 2.11
**9i**	12.33 ± 3.25	24.44 ± 0.90	13.34 ± 1.55	12.70 ± 4.91	14.34 ± 3.18
**10i**	8.28 ± 2.21	32.93 ± 1.36	21.65 ± 3.52	18.01 ± 2.82	24.38 ± 2.75
**11a**	n.a.	n.a.	n.a.	n.a.	n.a.
**11b**	52.66 ± 13.96	n.a.	n.a.	n.a.	n.a.
**11c**	29.61 ± 12.89	n.a.	83.17 ± 11.85	n.a.	n.a.
**11d**	43.55 ± 14.45	n.a.	92.16 ± 12.69	n.a.	n.a.
**11e**	16.15 ± 4.95	38.43 ± 2.61	33.72 ± 5.13	39.05 ± 7.14	37.45 ± 7.80
**11f**	3.09 ± 1.56	7.66 ± 2.08	19.31 ± 6.71	54.03 ± 15.97	38.84 ± 7.88
**11g**	4.26 ± 3.97	14.07 ± 4.77	7.23 ± 16.22	32.99 ± 18.89	45.30 ± 17.33
**11h**	13.81 ± 2.64	12.80 ± 1.38	15.79 ± 2.26	22.38 ± 4.31	12.40 ± 1.82
**11i**	11.25 ± 2.14	2.98 ± 1.38	5.29 ± 2.56	10.35 ± 2.71	13.09 ± 2.68
**11j**	7.15 ± 3.53	11.28 ± 0.93	12.39 ± 2.44	17.32 ± 2.29	12.26 ± 1.88
**12i**	15.13 ± 3.16	16.44 ± 1.57	15.13 ± 4.94	12.82 ± 4.03	18.36 ± 8.39
**13f**	56.11 ± 26.95	84.38 ± 26.06	44.49 ± 10.88	n.a.	n.a.
**13i**	n.a.	n.a.	n.a.	n.a.	n.a.
cisplatin	1.78 ± 1.22	3.02 ± 1.79	11.80 ± 3.87	10.30 ± 6.23	10.66 ± 3.30

Human cancer cell lines: MV4-11: biphenotypic B cell myelomonocytic leukemia, A549: lung cancer; LoVo: colon adenocarcinoma, MCF-7: breast cancer. BALB/3T3: normal mouse fibroblasts cell line; n.a.—not active in the range of concentrations used (up to 100 µM).

## Supplementary Materials

The following are available online at https://www.mdpi.com/1996-1944/13/21/5010/s1, Figure S1. 1H NMR spectrum of 4’-fluoro-N-((1S,4S,5R)-2-((S)-1-phenylethyl)-2-azabicyclo[3.2.1]octan-4-yl)-[1,1’-biphenyl]-4-sulfonamide, Figure S2. 13C NMR spectrum of 4’-fluoro-N-((1S,4S,5R)-2-((S)-1-phenylethyl)-2-azabicyclo[3.2.1]octan-4-yl)-[1,1’-biphenyl]-4-sulfonamide, Figure S3. 1H NMR spectrum of N-((1S,3S,4R)-2-azabicyclo[2.2.1]heptan-3-ylmethyl)-4’-fluoro-[1,1’-biphenyl]-4-sulfonamide, Figure S4. 13C NMR spectrum of N-((1S,3S,4R)-2-azabicyclo[2.2.1]heptan-3-ylmethyl)-4’-fluoro-[1,1’-biphenyl]-4-sulfonamide, Figure S5. 1H NMR spectrum of (1S,3R,4R)-N-((2-((S)-1-phenylethyl)-2-azabicyclo[2.2.1]heptan-3-yl)methyl) methanesulfonamide, Figure S6. 13C NMR spectrum of (1S,3R,4R)-N-((2-((S)-1-phenylethyl)-2-azabicyclo[2.2.1]heptan-3-yl)methyl) methanesulfonamide, Figure S7. 1H NMR spectrum of (1S,3R,4R)-N-((2-((S)-1-phenylethyl)-2-azabicyclo[2.2.1]heptan-3-yl)methyl) benzenesulfonamide, Figure S8. 13C NMR spectrum of (1S,3R,4R)-N-((2-((S)-1-phenylethyl)-2-azabicyclo[2.2.1]heptan-3-yl)methyl) benzenesulfonamide, Figure S9. 1H NMR spectrum of (1S,3R,4R)-4-methyl-N-((2-((S)-1-phenylethyl)-2-azabicyclo[2.2.1]heptan-3-yl)methyl) benzenesulfonamide, Figure S10. 13C NMR spectrum of (1S,3R,4R)-4-methyl-N-((2-((S)-1-phenylethyl)-2-azabicyclo[2.2.1]heptan-3-yl)methyl) benzenesulfonamide, Figure S11. 1H NMR spectrum of 4-fluoro-N-(((1S,3S,4R)-2-((S)-1-phenylethyl)-2-azabicyclo[2.2.1]heptan-3-yl)methyl)benzenesulfonamide, Figure S12. 13C NMR spectrum of 4-fluoro-N-(((1S,3S,4R)-2-((S)-1-phenylethyl)-2-azabicyclo[2.2.1]heptan-3-yl)methyl)benzenesulfonamide, Figure S13. 1H NMR spectrum of 2’-fluoro-N-(((1S,3S,4R)-2-((S)-1-phenylethyl)-2-azabicyclo[2.2.1]heptan-3-yl)methyl)-[1,1’-biphenyl]-4-sulfonamide, Figure S14. 13C NMR spectrum of 2’-fluoro-N-(((1S,3S,4R)-2-((S)-1-phenylethyl)-2-azabicyclo[2.2.1]heptan-3-yl)methyl)-[1,1’-biphenyl]-4-sulfonamide, Figure S15. 1H NMR spectrum of 4’-fluoro-N-(((1S,4S,5R)-2-((S)-1-phenylethyl)-2-azabicyclo[3.2.1]octan-4-yl)methyl)-[1,1’-biphenyl]-4-sulfonamide, Figu-re S16. 13C NMR spectrum of 4’-fluoro-N-(((1S,4S,5R)-2-((S)-1-phenylethyl)-2-azabicyclo[3.2.1]octan-4-yl)methyl)-[1,1’-biphenyl]-4-sulfonamide, Figure S17. 1H NMR spectrum of 4’-fluoro-N-(((1S,3R,4R)-2-((S)-1-phenylethyl)-2-azabicyclo[2.2.1]heptan-3-yl)methyl)-N-(((1S,4R)-2-((S)-1-phenylethyl)-2-azabicyclo[2.2.1]heptan-3-yl)methyl)-[1,1’-biphenyl]-4-sulfonamide, Figure S18. 13C NMR spectrum of 4’-fluoro-N-(((1S,3R,4R)-2-((S)-1-phenylethyl)-2-azabicyclo[2.2.1]heptan-3-yl)methyl)-N-(((1S,4R)-2-((S)-1-phenylethyl)-2-azabicyclo[2.2.1]heptan-3-yl)methyl)-[1,1’-biphenyl]-4-sulfonamide, Table S1: Experimental details for 9i and 10i, Table S2: Selected geometric parameters (Å, °) for 9i and 10i, Table S3: Selected hydrogen bond parameters.
