# Supplementary material for: Biaryl Sulfonamides Based on the 2-Azabicycloalkane Skeleton—Synthesis and Antiproliferative Activity
Source: Materials (Basel). 2020 Nov 6;13(21):5010. doi: 10.3390/ma13215010 (PMC7664408; doi:10.3390/ma13215010)
Supplement: Supplementary file 1 [file materials-13-05010-s001.zip › materials-968916-supplementary.docx]

Supporting Materials

Biaryl Sulfonamides Based on the 2-Azabicycloalkane Skeleton—Synthesis
and Antiproliferative Activity

Dominika Iwan ^1^, Karolina Kamińska ^1^, Elżbieta Wojaczyńska ^1,^*, Mateusz Psurski ^2^,
Joanna Wietrzyk ^2^ and Marek Daszkiewicz ^3^

^1^ Faculty of Chemistry, Wrocław University of Science and Technology, Wybrzeże Wyspiańskiego 27, 50-370 Wrocław, Poland; dominika.iwan@pwr.edu.pl (D.I.), karolina.kaminska@pwr.edu.pl (K.K.)

^2^ Department of Experimental Oncology, Hirszfeld Institute of Immunology and Experimental Therapy, Polish Academy of Sciences, Rudolfa Weigla 12, 53-114 Wrocław, Poland; mateusz.psurski@hirszfeld.pl (M.P.); joanna.wietrzyk@hirszfeld.pl (J.W.)

^3^ Institute of Low Temperature and Structure Research, Polish Academy of Sciences, Okólna St. 2, 50-422 Wrocław, Poland; marek.daszkiewicz@intibs.pl

***** Correspondence: elzbieta.wojaczynska@pwr.edu.pl; Tel.: + 48-71-3202410

Received: 30 September 2020; Accepted: 3 November 2020; Published: date

Preparation of Starting Compounds

General Procedure of Synthesis of Bicyclic Amines

The bicyclic isomeric amines, based on 2-azabicyclo[2.2.1]heptane and 2-azabicyclo[3.2.1]octane skeletons **4** and **6** as well as dimeric form possessing two bicyclic units were prepared using the procedure described previously.[1]

A. ((1R,2R,4S,5S)-4-((R)-1-phenylethyl)bicyclo[3.2.1]octan-2-yl)methanamine (8)

Dissolved in ethanol (15 mL) (1*S*,4*S*,5*R*)-4-chloro-2-((S)-1-phenylethyl)-2-azabicyclo[3.2.1]octane (1.0 mmol; 249.8 mg) was added dropwise to a solution of sodium cyanide (2.4 mmol; 117.6 mg) in water (10 mL). The mixture was refluxed for 34 h. After that, 10% aqueous solution of sodium hydroxide and ethanol were added. Subsequently, resulting mixture was extracted with diethyl ether. Combined organic phases were dried over Na2SO4, filtered through the Celite® and concentrated under the vacuum. The residue was chromatographed on silica (eluent: *n*-hexane: ethyl acetate 3:1 v/v), yielding (1*R*,2*R*,4*S*,5*S*)-4-(1-phenylethyl)bicyclo[3.2.1]octane-2-carbonitrile **7**.

The solution of (1*R*,2*R*,4*S*,5*S*)-4-(1-phenylethyl)bicyclo[3.2.1]octane-2-carbonitrile **7** (1.0 mmol, 240.3 mg) in dry THF (10 mL) was added dropwise in an ice bath to a suspension of lithium aluminum hydride (3.0 mmol, 114.0 mg) in dry THF (15 mL). The reaction mixture was stirred overnight. 10% aqueous solution of sodium hydroxide was added and then crude residue isolated form inorganic salts was extracted with dichloromethane. The organic phases were dried over Na2SO4, filtered through the Celite® and evaporated under vacuum. Resulted ((1*R*,2*R*,4*S*,5*S*)-4-(1-phenylethyl)bicyclo[3.2.1]octan-2-yl)methanamine **8** was purified by column chromatography on silica (eluent: dichloromethane: methanol 95: 5 v/v) with 64% yield.

Yellow oil; 156 mg; 64% yield. 1H NMR (CDCl_3_, 400 MHz): δ 7.32–7.26 (m, 4H, ArH), 7.21–7.16 (m, 1H, ArH), 3.58–3.55 (t, *J* = 4.4 Hz, 1H), 3.33–3.28 (q, *J* = 6.8 Hz, 1H), 2.82–2.72 (s, *J* = 6,0 Hz, 2H), 2.31–2.28 (m, 1H), 2.21–2.18 (q, *J* = 4.4 Hz, 1H), 2.13-2.08 (m, 1H), 1.86–1.83 (m, 1H), 1.78–1.71 (m, 2H), 1.56 (br s, 3H), 1.39–1.27 (m, 5H), 1.24–1.18 (m, 1H). 13C NMR (CDCl_3_, 125 MHz): δ 146.3, 128.4, 127.3, 126.7, 62.8, 56.2, 46.8, 44.3, 43.8, 36.7, 34.3, 29.8, 21.8, 21.7 ppm. HRMS (ESI-TOF) m/z: found [M + H]^+^ 245.2014; calc. for [C_19_H_24_N_2_]^+^: 245.2018 4

B. (1S,3R,4R)-tert-butyl 3-(aminomethyl)-2-azabicyclo[2.2.1]heptane-2-carboxylate (5)

Amine **5** was prepared using modified procedure described by Jin et al. [2]

A solution of (1*R*,3*S*,4*S*)-ethyl 2-(1-phenylethyl)-2-azabicyclo[2.2.1]hept-5-ene-3-carboxylate **1** (1.0 mmol, 271.0 mg) in absolute ethanol (15 mL) was stirred with palladium on carbon (10% w/w, 27.1 mg) and hydrogen (under pressure 7.0 bar) in reactor for 24 h. Subsequently, the mixture was filtered through the Celite® and concentrated under the vacuum. In case if conversion was not quantitative, the residue was chromatographed on silica (eluent: *n*-hexane: ethyl acetate 1:1 v/v), leading to the product with reduced double bond in the bicyclic ring and secondary amine group in position 2.

Reduced (1*S*,3*S*,4*R*)-ethyl 2-azabicyclo[2.2.1]heptane-3-carboxylate (1.0 mmol, 169.2 mg) was dissolved in dichloromethane (20 mL) in an atmosphere of an inert gas. TEA (1.0 mmol, 101.2 mg) and di-*tert*-butyl dicarbonate (1.1 mmol, 240.1 mg) were added. The mixture was stirred in room temperature for 18 h. After that dichloromethane and water was added and reacting mixture was extracted 3 times. Combined organic phases were dried over Na2SO4, filtered through the Celite® and evaporated under vacuum. The residue was chromatographed on silica (eluent:*n*-hexane:ethyl acetate 1:1 v/v), yielding the product with a Boc-protected amine group.

Further, the protected (1*S*,3*S*,4*R*)-2-tert-butyl 3-ethyl 2-azabicyclo[2.2.1]heptane-2,3-dicarboxylate (1.0 mmol, 269.3 mg ) was dissolved in dry THF (10 mL) and a suspension of lithium aluminum hydride (2.0 mmol, 76.0 mg) in dry THF (10 mL) was added dropwise in an ice bath. After 1.5 h 10% aqueous solution of sodium hydroxide was added dropwise. Inorganic salts were removed and a reaction mixture was extracted 3 times with dichloromethane, organic phases were dried over Na2SO4, filtered through the Celite® and evaporated under vacuum. Crude product **3** was proceed without further purification.

In atmosphere of an inert gas alcohol (1*S*,3*R*,4*R*)-tert-butyl 3-(hydroxymethyl)-2-azabicyclo[2.2.1]heptane-2-carboxylate **3** (1.0 mmol, 227.3 mg) and triphenylphosphine was dissolved in toluene (15 mL). Hydrazoic acid (1.3 mmol, 1.3 mL) was added dropwise in temperature of 0–5 °C. Subsequently, 40% solution of diethyl azodicarboxylate (1.5 mmol, 0.6 mL) in toluene was added dropwise within about 1 h. The mixture was stirred for 18 h and then alkalized with 10% aqueous sodium hydroxide solution. The solvent was evaporated 5 under the vacuum and the residue was extracted 3 times with diethyl ether. Crude product was chromatographed on silica (eluent:*n*-hexane:ethyl acetate 1:1 v/v), yielding the desired azide.

A solution of (1*S*,3*R*,4*R*)-tert-butyl 3-(azidomethyl)-2-azabicyclo[2.2.1]heptane-2-carboxylate (1.0 mmol, 252.3 mg) in absolute ethanol (15 mL) was stirred with palladium on carbon (10% w/w, 25.2 mg) and hydrogen (under pressure 3.5 bar) in reactor for 24 h. Subsequently, the mixture was filtered through the Celite® and concentrated under the vacuum, yielding pure product (1S,3R,4R)-tert-butyl 3-(aminomethyl)-2-azabicyclo[2.2.1]heptane-2-carboxylate **5**.

Yellowish oil; 219.5 mg; 97% yield. Mixture of conformers:1H NMR (CDCl_3_, 400 MHz):δ 3.99 (s, 1H), 3.17–3.14 (m, 1H), 2.86–2.81 (dd, *J* = 8.0 Hz; *J =* 5.2 Hz, 1 H), 2.50–2.34 (m, 2H), 1.70–1.61 (m, 5H), 1.54–1.51 (m, 1H), 1.40 (s, 9H), 1.19–1.16 (m, 1H) ppm. 13C NMR (CDCl_3_, 125 MHz): δ 156.2, 79.54, 66.7, 57.9, 45.5, 39.6, 35.0, 30.2, 28.6, 27.7 ppm. HRMS (ESI-TOF) m/z: found [M + H]^+^ 227.1760; calc. for [C_12_H_22_N_2_O_2_]^+^:227.1759

General Procedure Of The Synthesis Of Sulfonamides

All of the chiral sulfonamides based on 2-azabicycloalkane scaffold with an exception of sulfonamide **10i** were synthesized as described in our previous paper. [3] The appropriate sulfonyl chloride (1.0 mmol) was added to a solution of primary amine **4**, **6** or **8** (1.0 mmol) and powdered potassium hydroxide (1.8 mmol) in a dry dichloromethane(15 mL), which was stirred at room temperature. After 24 h, the resulting reaction mixture was poured over brine and extracted with dichloromethane. The organic phases were dried over Na2SO4, filtered through the Celite®, washed with dichloromethane, and evaporated under vacuum. The resulting residue was purified by column chromatography on silica (eluent: ethyl acetate/n-hexane 1:1 v/v) ,yielding sulfonamide **9** (from amine **4**), **11**(from amine **6**) and **12** (from amine **8**) with general yield 46–83%.

Spectral Data

Compounds **9f**, **9h**, **11e**–**11i**, **13f** have been synthesized, characterized and described by our group in previous work. [3] 6

4. ‘-fluoro-N-((1S,4S,5R)-2-((S)-1-phenylethyl)-2-azabicyclo[3.2.1]octan-4-yl)-[1,1’-biphenyl]-4-sulfonamide (9i)

Colorless solid; 180 mg; 76% yield; mp. 139–140 °C; [α]D20 = −18.0 (*c* 0.7; CH_2_Cl_2_). 1H NMR (CDCl_3_, 400 MHz): δ 7.66–7.64 (m, 2H, ArH), 7.51–7.48 (m, 2H, ArH), 7.43–7.41 (m, 2H, ArH), 7.25–7.21 (m, 3H, ArH), 7.18–7.14 (m, 4H, ArH), 5.04 (m, 1H), 3.53 (br s, 1H), 3.18-3.17 (m, 1H), 3.02 (br s, 1H), 2.30 (m, 1H), 2.07–2.04 (m, 1H), 1.86–1.84 (m, 2H), 1.60 (m, 3H), 1.32–1.22 (m, 5H) ppm. 13C NMR (CDCl_3_, 125 MHz): δ 164.4, 161.9, 144.0, 135.5 (d, *J* = 14.0 Hz), 129.0 (d, *J* = 32.0 Hz), 128.5, 127.4 (d, *J* = 20.4), 127.2 (overlapped), 127.0 (overlapped), 116.2–116.0 (d, *J* = 86.8 Hz), 62.3, 55.7, 52.7, 49.2, 40.0, 34.4, 27.2, 21.7, 21.3 ppm. IR (KBr): 3436, 3294, 1927, 1601, 1520, 1491, 1319, 1043, 823, 664 cm^−1^. HRMS (ESI-TOF) m/z: found [M + H]^+^ 465.2016; calc. for [C_27_H_29_FN_2_O_2_S]^+^: 465.2012


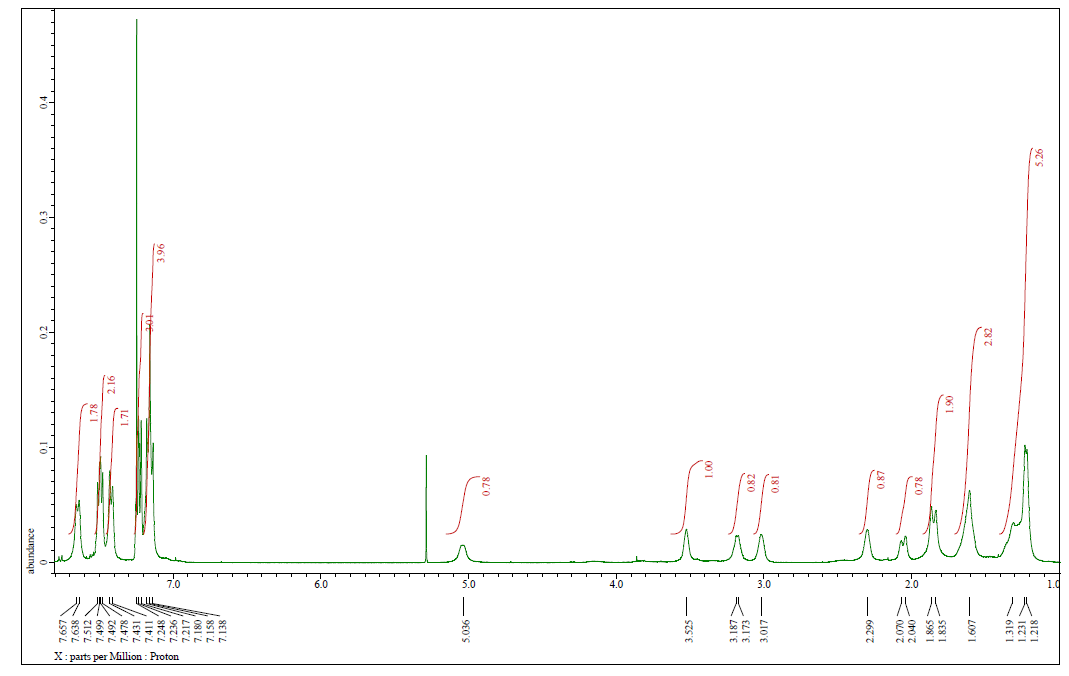


**Figure S1.** 1H NMR spectrum of 4’-fluoro-N-((1S,4S,5R)-2-((S)-1-phenylethyl)-2-azabicyclo[3.2.1]octan-4-yl)-[1,1’-biphenyl]-4-sulfonamide 7.


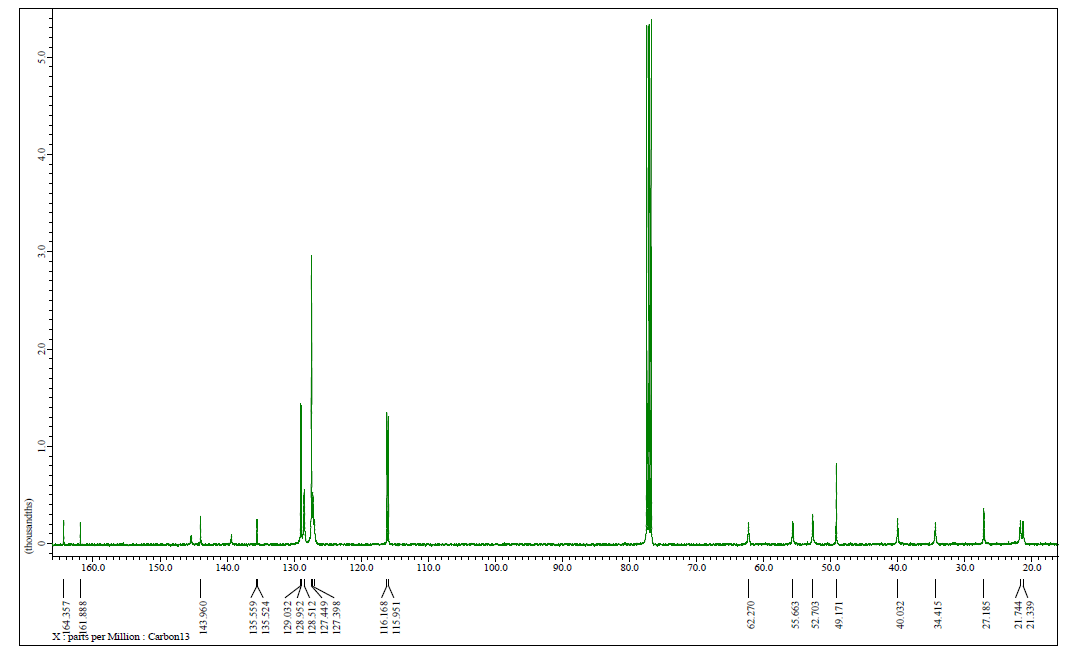


**Figure S2.** 13C NMR spectrum of 4’-fluoro-N-((1S,4S,5R)-2-((S)-1-phenylethyl)-2-azabicyclo[3.2.1]octan-4-yl)-[1,1’-biphenyl]-4-sulfonamide.

N-((1S,3S,4R)-2-azabicyclo[2.2.1]heptan-3-ylmethyl)-4’-fluoro-[1,1’-biphenyl]-4-sulfonamide (10i)

Procedure: The 4’-fluorobiphenyl-4-sulfonyl chloride (1.0 mmol, 270.7 mg) was added to a solution of primary amine **5** (1.0 mmol, 226.3 mg) and powdered potassium hydroxide (1.8 mmol, 101.0 mg) in a dry dichloromethane(15 mL), which was stirred at room temperature. After 24 h, the resulting reaction mixture was poured over brine and extracted with dichloromethane. The organic phases were dried over Na2SO4, filtered through the Celite®, washed with dichloromethane, and evaporated under vacuum. The resulting residue was purified by column chromatography on silica (eluent: dichloromethane) and crystallized from *n*-hexane: dichloromethane, yielding sulfonamide **10i**.

Colorless solid; 8 mg; 46% yield; mp. 155 °C; [α]D20 = −20.9 (*c* 0.4; CH_2_C_l2_). 1H NMR (CDC_l3_, 400 MHz): δ 7.91–7.88 (m, 2H, ArH), 7.66–7.62 (m, 2H, ArH), 7.57–7.53 (m, 2H, ArH), 7.18–7.12 (m, 2H, ArH), 3.72 (br s, 2H), 3.50 (s, 1H), 3.04–2.98 (m, 2H), 2.62–2.56 (m, 1H), 2.19 (m, 1H), 1.70–1.57 (m, 2H), 1.52–1.50 (m, 1H), 1.45–1.34 (m, 2H), 1.21–1.18 (m, 1H) ppm. 13C NMR (CDC_l3_, 125 MHz): δ 164.4, 161.9, 144.4, 138.7, 135.6 (d, *J* = 14.0 Hz), 129.1–129.0 (d, *J* = 25.2 Hz), 127.7–127.6 (d, *J* = 7.8 Hz), 116.2–166.0 (d, *J* = 84.4 Hz), 60.7, 56.5, 47.3, 39.4, 834.7, 31.8, 28.4 ppm. IR (KBr): 3436, 3319, 1606, 1520, 1326, 1158, 1097, 816, 632 cm^−1^. HRMS (ESI-TOF) m/z: found [M + H]^+^ 361.1386; calc. for [C_19_H_21_FN_2_O_2_S]^+^: 361.1386


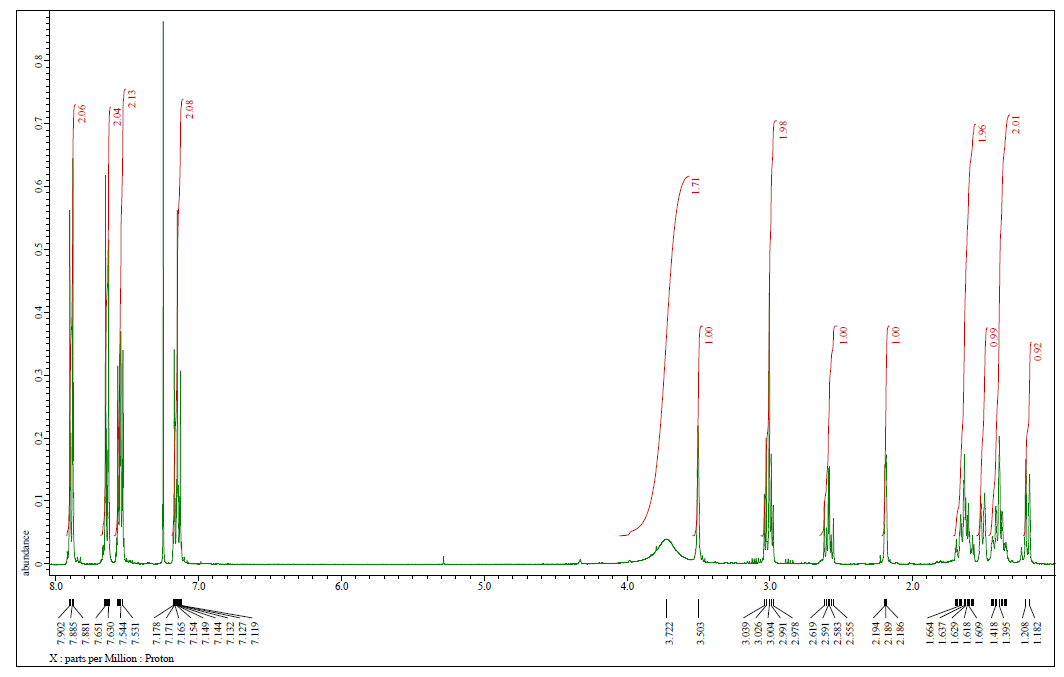


**Figure S3.** 1H NMR spectrum of N-((1S,3S,4R)-2-azabicyclo[2.2.1]heptan-3-ylmethyl)-4’-fluoro-[1,1’- biphenyl]-4-sulfonamide.


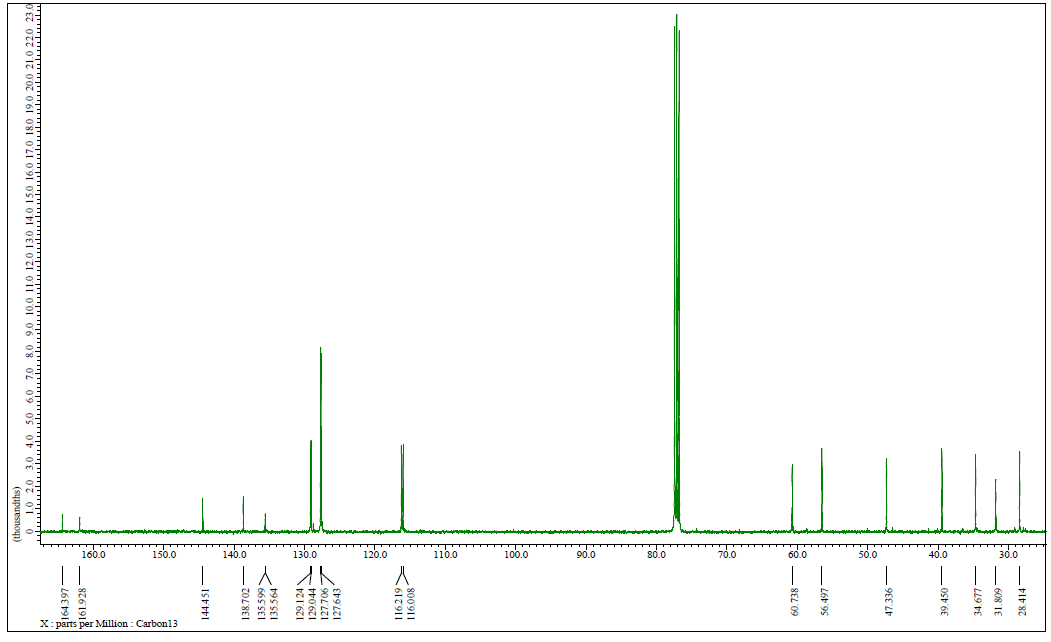


**Figure S4.** 13C NMR spectrum of N-((1S,3S,4R)-2-azabicyclo[2.2.1]heptan-3-ylmethyl)-4’-fluoro-]1,1’-biphenyl]-4-sulfonamide 9.

(1S,3R,4R-N-((2-((S)-1-phenylethyl)-2-azabicyclo[2.2.1]heptan-3-yl)methyl) methanesulfonamide (11a)

Yellow oil; 60 mg; 20% yield; [α]D20 = −8.01 (*c* 0.17, CH_2_Cl_2_). 1H NMR (400 MHz, CDCl_3_): δ 7.63–7.60 (m, 2H, ArH), 7.45–7.39 (m, 3H, ArH), 5.56 (br s, 1H), 4.14 (s, 1H), 3.82–3.79 (m, 1H), 2.70 (s, 1H), 2.61 (s, 3H), 2.56 (m, 1H), 2.44–2.36 (m, 2H), 2.08 (s, 2H), 1.88–1.80 (m, 1H), 1.67–1.65 (d, *J* = 6.8 Hz, 3H), 1.57–1.55 (m, 2H) ppm. 13C NMR (125 MHz, CDCl_3_): δ 146.2, 128.5, 128.2, 127.2, 67.2, 60.8, 59.2, 49.6, 43.1, 42.3, 38.8, 34.8, 28.6, 23.0 ppm. IR (KBr): 3283, 2968, 1720, 1454, 1316, 1151, 703, 523 cm^−1^. HRMS (ESI-TOF) m/z: found [M + H]^+^ 309.1638; calcd. for [C_16_H_25_N_2_O_2_S]^+^: 309.1637.


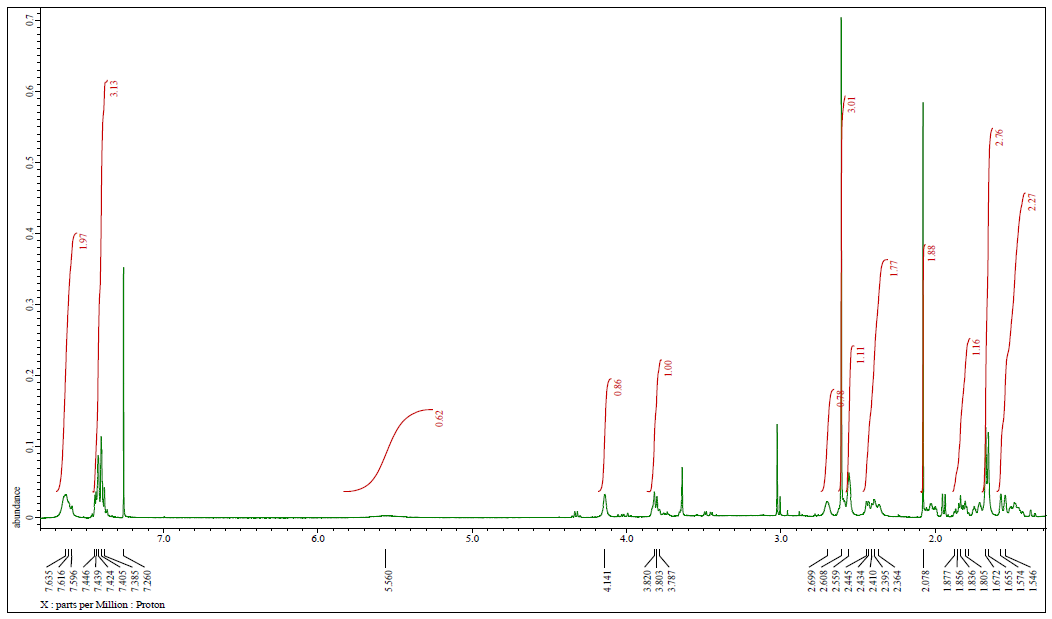


**Figure S5.** 1H NMR spectrum of (1S,3R,4R-N-((2-((S)-1-phenylethyl)-2-azabicyclo[2.2.1]heptan-3-yl)methyl) methanesulfonamide


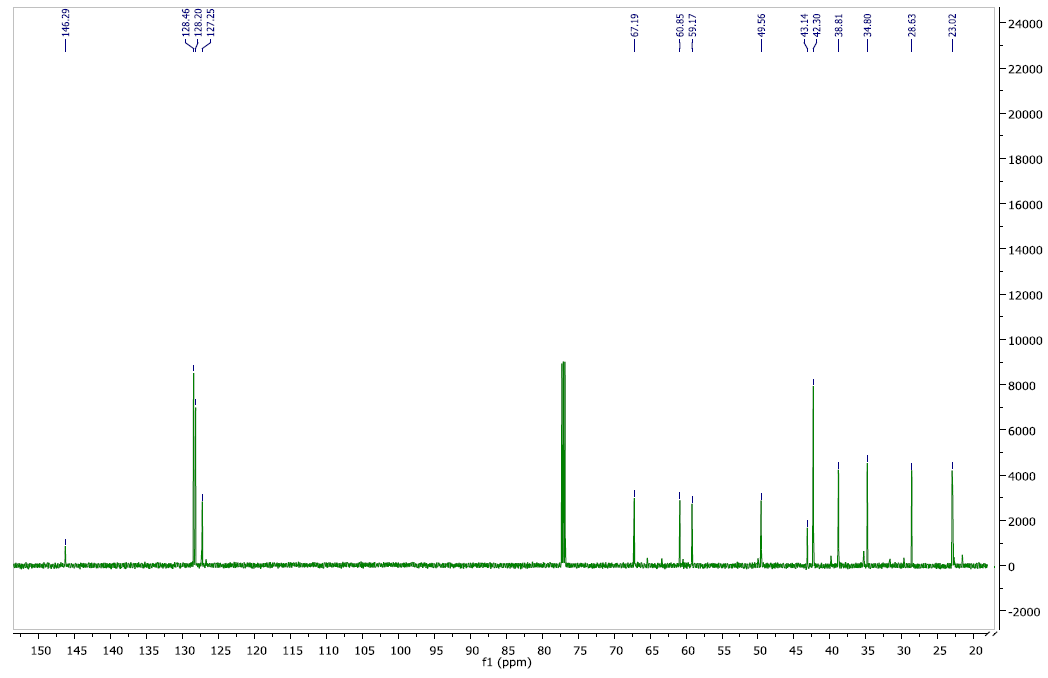


**Figure S6.** 13C NMR spectrum of (1S,3R,4R-N-((2-((S)-1-phenylethyl)-2-azabicyclo[2.2.1]heptan-3-yl)methyl) methanesulfonamide.

(1S,3R,4R)-N-((2-((S)-1-phenylethyl)-2-azabicyclo[2.2.1]heptan-3-yl)methyl) benzenesulfonamide (11b)

White solid; 160 mg; 43% yield; mp 107–109 °C; [α]D20 = +15.3 (*c* 0.68, CH_2_Cl_2_). 1H NMR (400 MHz, CDCl_3_): δ 7.57–7.54 (m, 3H, ArH), 7.45–7.42 (m, 2H, ArH), 7.17–7.16 (m, 2H, ArH), 7.05–7.04 (m, 3H, ArH), 4.22 (s, 1H), 3.58 (s, 1H), 3.41–3.37 (q, *J* = 4.4 Hz, 1H), 2.18–2.16 (d, *J* = 8.0 Hz, 1H), 2.12 (d, *J* = 4.2 Hz, 1H), 2.02–2.01 (d, *J* = 8.4 Hz, 1H), 1.87–1.85 (m, 2H), 1.81–1.79 (d, *J* = 10.2 Hz, 1H), 1.58 (m, 1H), 1.35 (m, 1H), 1.27 (d, *J* = 1.2 Hz, 3H), 1.20–1.18 (m, 2H) ppm. 13C NMR (125 MHz, CDCl_3_): δ 145.7, 139.7, 132.2, 128.8, 128.3, 127.5, 127.4, 126.9, 67.6, 60.8, 58.7, 47.0, 41.8, 35.6, 29.4, 22.5, 22.2 ppm. IR (KBr): 3323, 2978, 2876, 1449, 1337, 1166, 691, 574 cm^−1^. HRMS (ESI-TOF) m/z: found [M + H]^+^ 371.1794; calcd. for [C_21_H_27_N_2_O_2_S]^+^: 371.1793 11


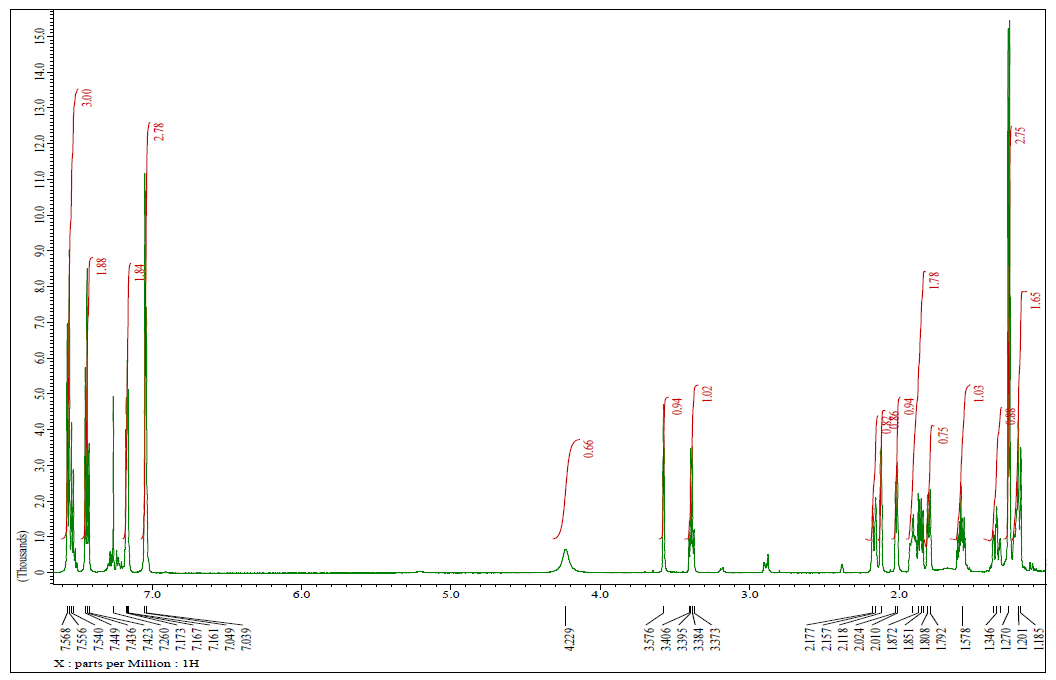


**Figure S7.** 1H NMR spectrum of (1S,3R,4R)-N-((2-((S)-1-phenylethyl)-2-azabicyclo[2.2.1]heptan-3- yl)methyl) benzenesulfonamide.


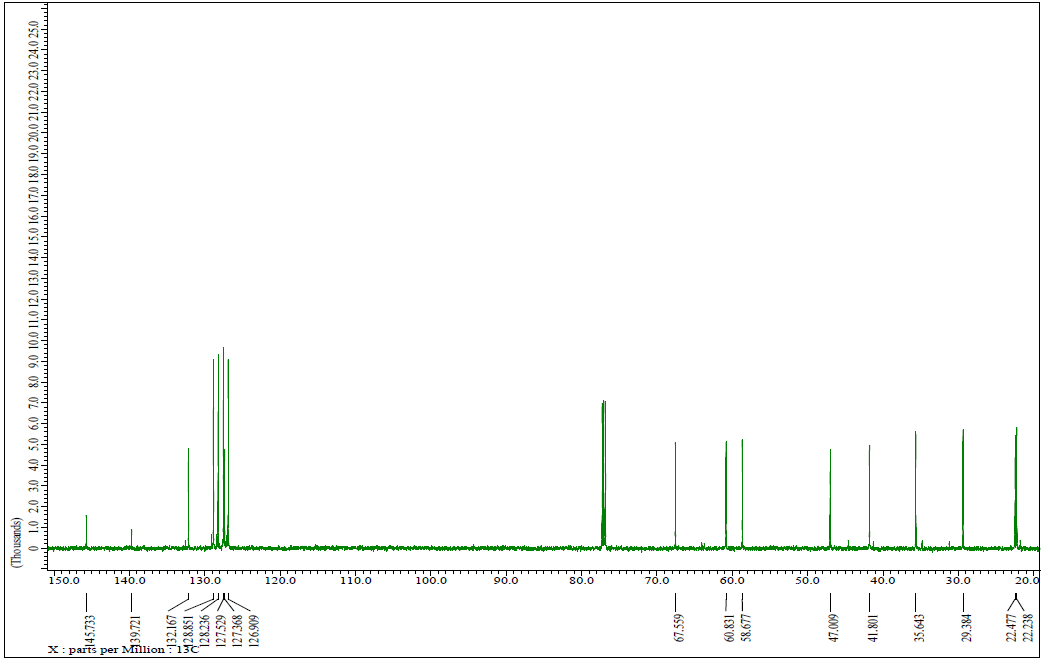


**Figure S8.** 13C NMR spectrum of (1S,3R,4R)-N-((2-((S)-1-phenylethyl)-2-azabicyclo[2.2.1]heptan-3- yl)methyl) benzenesulfonamide 12.

(1S,3R,4R)-4-methyl-N-((2-((S)-1-phenylethyl)-2-azabicyclo[2.2.1]heptan-3-yl)methyl) benzenesulfonamide (11c)

Yellow solid; 150 mg; 61% yield; mp 124–127 oC; [α]D20 = +28.2 (*c* 0.24, CH_2_C_l2_). 1H NMR (400 MHz, CDCl_3_): δ 7.44–7.43 (m, 2H, ArH), 7.26–7.22 (m, 2H, ArH), 7.18–7.16 (m, 2H, ArH), 7.06–7.05 (m, 3H, ArH), 4.14 (br s, 1H), 3.57 (s, 1H), 3.41–4.37 (q, *J* = 4.4 Hz, 1H), 2.44 (s, 3H), 2.13 (m, 2H), 2.02–2.01 (m, 1H), 1.91 (m, 2H), 1.86 (m, 2H), 1.81–1.79 (m, 1H), 1.59 (m, 1H), 1.40–1.34 (m, 1H), 1.27–1.26 (d, *J* = 4.4 Hz, 3H), 1.20–1.18 (m, 2H) ppm. 13C NMR (125 MHz, CDCl_3_): δ 145.8, 142.9, 136.8, 129.4, 128.2, 127.5, 127.2, 127.0, 67.5, 60.8, 58.6, 46.9, 41.7, 35.6, 29.5, 22.5, 22.2, 21.5 ppm. IR (KBr): 3249, 2957, 1452, 1328, 1161, 708, 553 cm^−1^. HRMS (ESI-TOF) m/z: found [M + H]^+^ 385.1942; calcd. for [C_22_H_29_N_2_O_2_S]^+^: 385.1950.


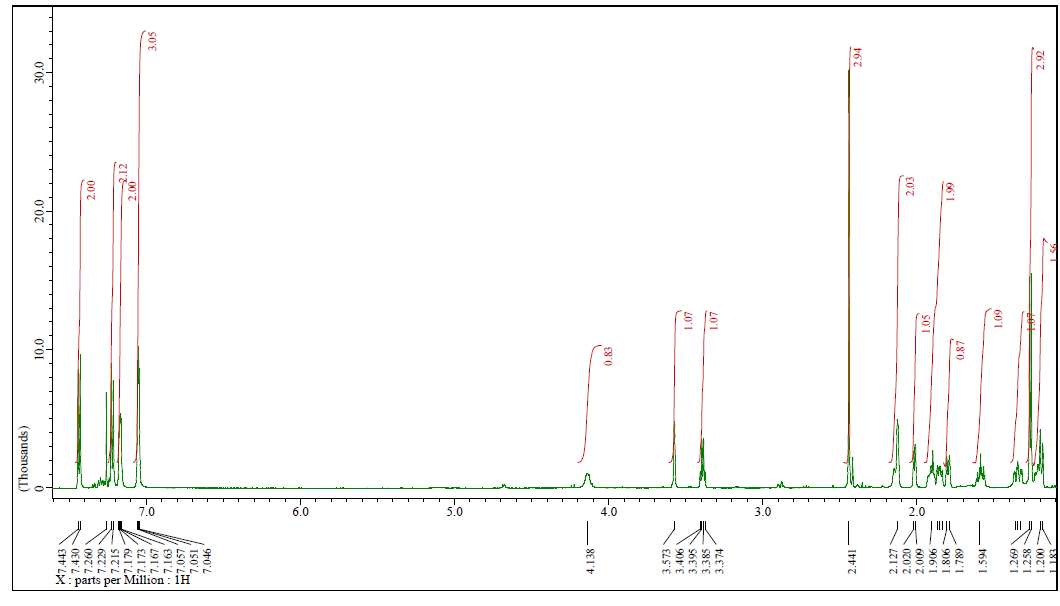


**Figure S9.** 1H NMR spectrum of (1S,3R,4R)-4-methyl-N-((2-((S)-1-phenylethyl)-2-azabicyclo[2.2.1]heptan-3-yl)methyl) benzenesulfonamide 13.


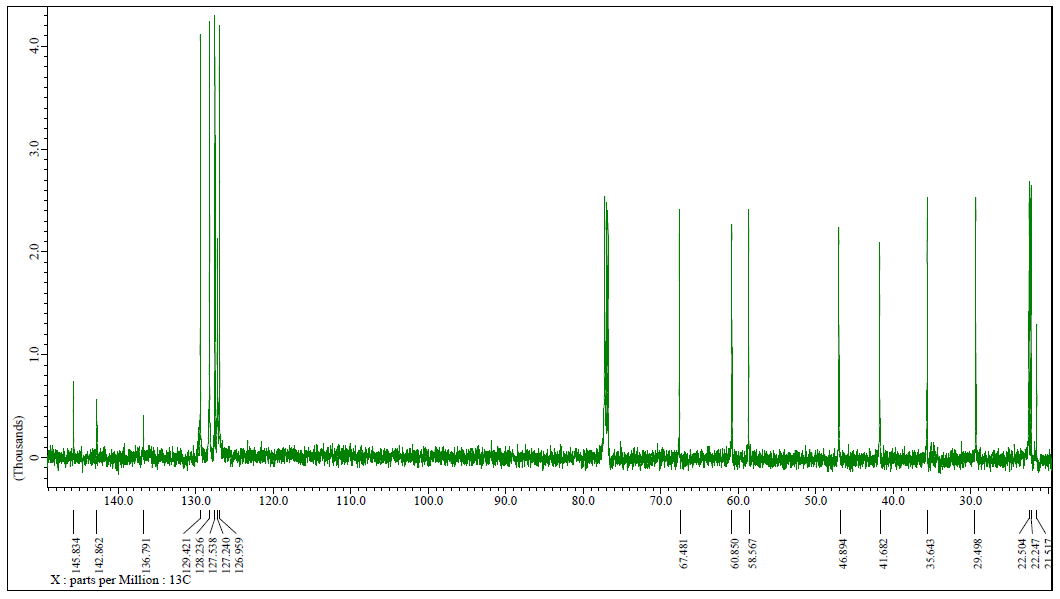


**Figure S10.** 13C NMR spectrum of (1S,3R,4R)-4-methyl-N-((2-((S)-1-phenylethyl)-2-azabicyclo[2.2.1]heptan-3-yl)methyl) benzenesulfonamide.

4. -fluoro-N-(((1S,3S,4R)-2-((S)-1-phenylethyl)-2-azabicyclo[2.2.1]heptan-3-yl)methyl)benzenesulfonamide (11d)

White solid; 170 mg; 72% yield; mp. 105–107 °C; [α]D20 = + 52.5 (*c* 0.7; CH_2_Cl_2_). 1H NMR(CDCl_3_, 400 MHz): δ 7.56–7.54 (m, 2H, ArH), 7.18–7.15 (m, 2H, ArH), 7.12–7.05 (m, 5H, ArH), 4.19 (br s, 1H), 3.57 (s, 1H), 3.41–3.36 (q, *J* = 6.4 Hz, 1H), 2.14–2.10 (m, 2H), 2.02 (dd, *J =* 4.4 Hz, *J* = 2.0 Hz, 1H), 1.94–1.86 (m, 1H), 1.84–1.77 (m, 2H), 1.61–1.56 (m, 1H), 1.37–1.19 (m, 6H) ppm. 13C NMR(CDCl_3_, 125 MHz): δ 166.2, 163.6, 145.9, 135.8, 129.7–129.6 (d, *J* = 40.0 Hz), 128.3, 127.6–127.4 (d, *J* = 80.0 Hz), 116.2–116.0 (d, *J* = 80.0 Hz), 67.6, 60.9, 58.8, 47.0, 41.8, 35.7, 29.4, 22.5 (overlapped), 22.3 ppm. IR (KBr): 3436, 3257, 2961, 1903, 1593, 1495, 1153, 1055, 837, 711 cm^−1^. HRMS (ESI-TOF) m/z: found [M + H]^+^ 389.1689; calc. for [C_21_H_25_FN_2_O_2_S]^+^: 389.1699 14


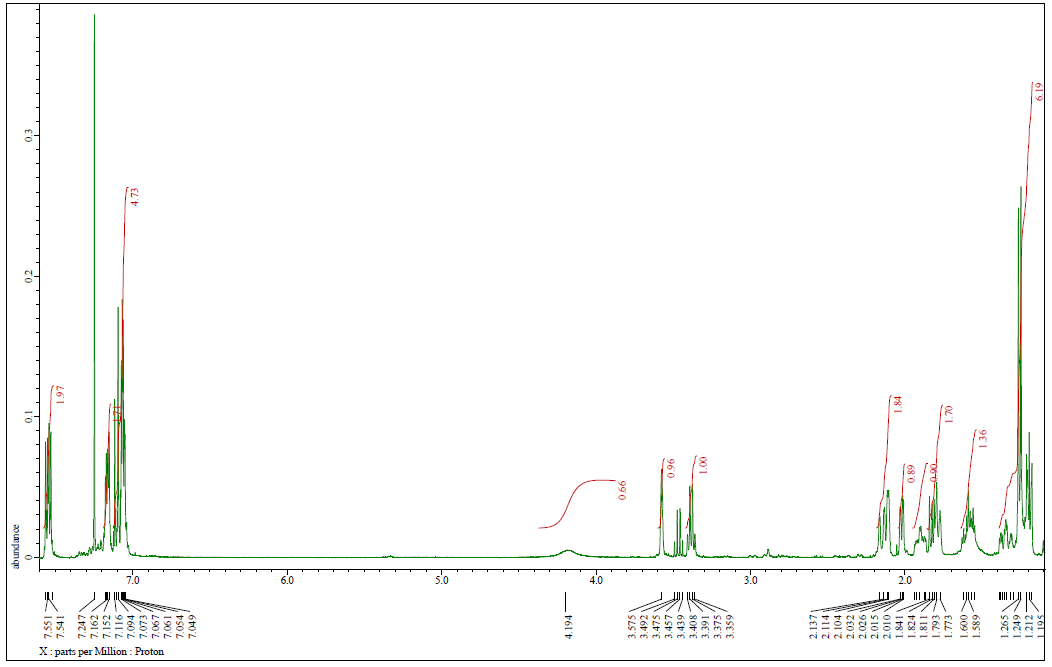


**Figure S11.** 1H NMR spectrum of 4-fluoro-N-(((1S,3S,4R)-2-((S)-1-phenylethyl)-2- azabicyclo[2.2.1]heptan-3-yl)methyl)benzenesulfonamide.


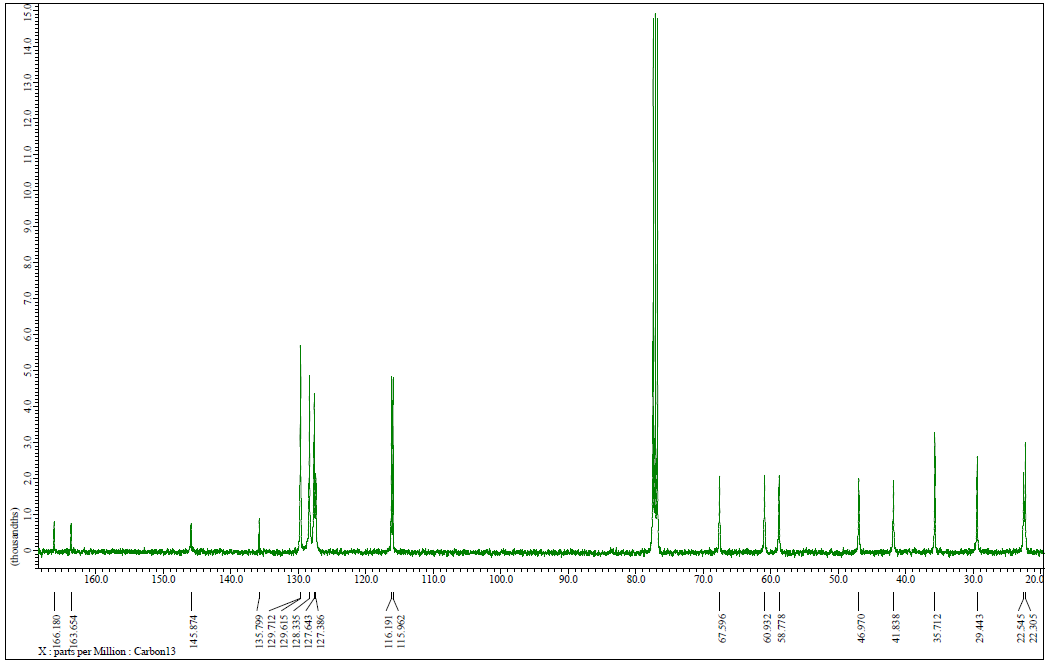


**Figure S12.** 13C NMR spectrum of 4-fluoro-N-(((1S,3S,4R)-2-((S)-1-phenylethyl)-2- azabicyclo[2.2.1]heptan-3-yl)methyl)benzenesulfonamide 15.

2. ‘-fluoro-N-(((1S,3S,4R)-2-((S)-1-phenylethyl)-2-azabicyclo[2.2.1]heptan-3-yl)methyl)-[1,1’-biphenyl]-4-sulfonamide (11j)

White solid; 150 mg; 78% yield; mp. 95 °C; [α]D20 = + 16.1 (*c* 0.6; CH2Cl2). 1H NMR(CDCl3, 400 MHz): δ 7.61 (m, 4H, ArH), 7.47–7.37 (m, 2H, ArH), 7.29–7.15 (m, 4H, ArH), 7.02–7.00 (m, 3H, ArH), 4.27 (m, 1H), 3.58 (s, 1H), 3.41–3.36 (q, *J* = 6.4, 1H), 2.24–2.21 (m, 1H), 2.14 (m, 1H), 2.04–2.03 (m, 1H), 1.90–1.82 (m, 3H), 1.63–1.55 (m, 1H), 1.38–1.19 (m, 6H) ppm. 13C NMR (CDCl_3_, 125 MHz): δ 161.1, 158.6, 145.8, 139.9, 138.7, 130.7 (d, *J* = 11.6 Hz), 130.2 (d, *J* = 32.0 Hz ), 129.6–129.5 (d, *J* = 11.6 Hz), 128.3, 127.5 (m), 127.1, 124.8 (d, *J* = 14.0 Hz), 116.6, 116.4, 67.5, 60.9, 58.7, 47.1, 42.0, 35.8, 29.5, 22.5, 22.3 ppm. IR (KBr): 3276, 2969, 2871, 1956, 1453, 1322, 1161, 1093, 823, 757, 702, 657 cm^−1^. HRMS (ESI-TOF) m/z: found [M + H]^+^ 465.2011; calc. for [C_27_H_33_N_3_O_2_S]^+^: 465.2012


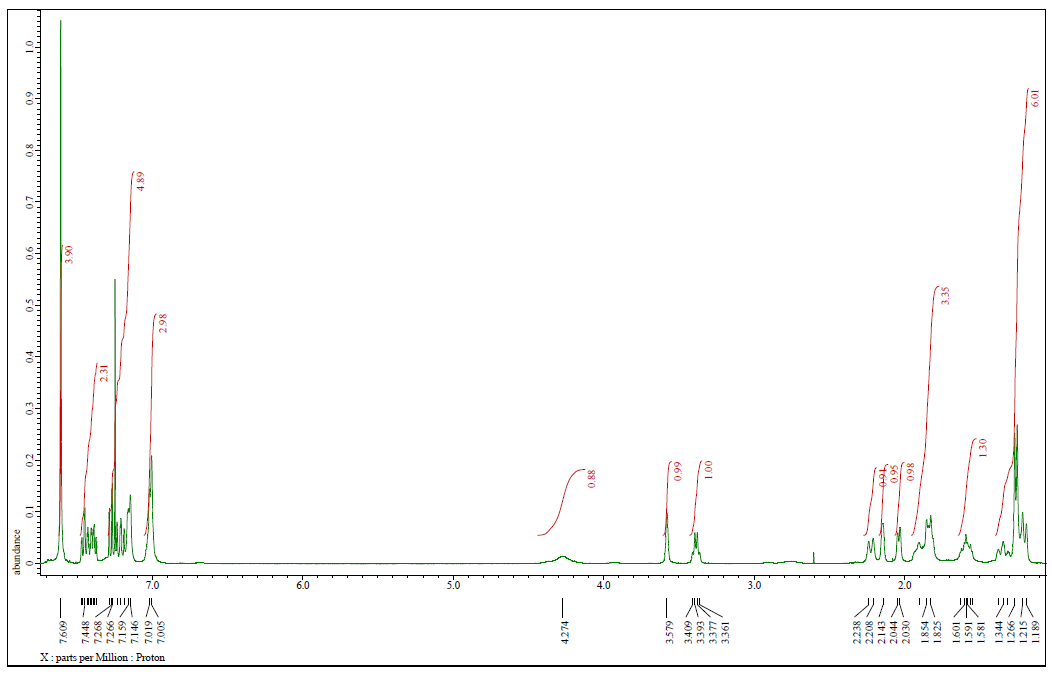


**Figure S13.** 1H NMR spectrum of 2’-fluoro-N-(((1S,3S,4R)-2-((S)-1-phenylethyl)-2-azabicyclo[2.2.1]heptan-3-yl)methyl)-[1,1’-biphenyl]-4-sulfonamide 16.


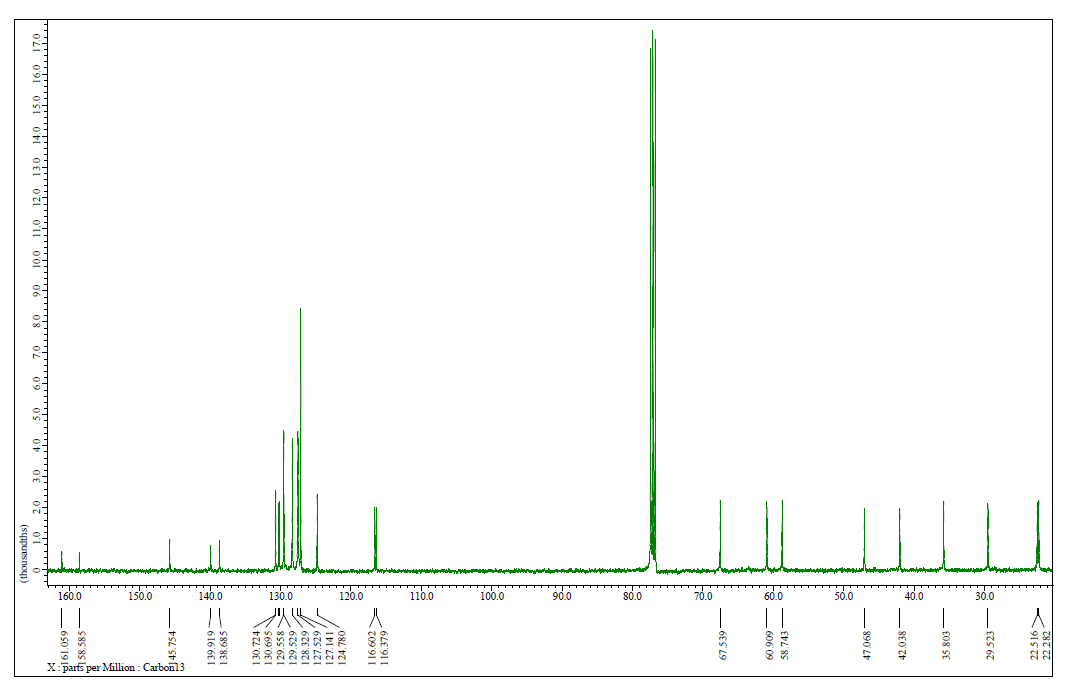


**Figure S14.** 13C NMR spectrum of 2’-fluoro-N-(((1S,3S,4R)-2-((S)-1-phenylethyl)-2-azabicyclo[2.2.1]heptan-3-yl)methyl)-[1,1’-biphenyl]-4-sulfonamide.

4. ‘-fluoro-N-(((1S,4S,5R)-2-((S)-1-phenylethyl)-2-azabicyclo[3.2.1]octan-4-yl)methyl)-[1,1’-biphenyl]-4-sulfonamide (12i)

White solid; 180 mg; 77% yield; mp. 128–129 °C; [α]D20 = + 29.4 (*c* 0.7; CH_2_Cl_2_). 1H NMR (CDCl_3_, 600 MHz): δ 7.83 (m, 2H, ArH), 7.65–7.59 (m, 4H, ArH), 7.55–7.30 (m, 4H, ArH), 7.28–7.26 (m, 1H, ArH), 7.22–7.18 (m, 2H, ArH) 5.48 (br s, 1H), 3.61 (s, 1H), 3.32–3.31 (m, 1H), 3.14–3.13 (m, 1H), 3.08–3.05 (m, 1H), 2.27–2.19 (m, 3H), 1.19–1.92 (m, 1H), 1.73–1.72 (m, 2H), 1.46 (s, 1H), 1.29–1.24 (m, 6H) ppm. 13C NMR (CDCl_3_, 125 MHz): δ 163.9, 162.3, 144.2, 138.7, 135.6, 129.0 (d, *J* = 30.0 Hz), 128.5, 127.7, 127.4, 127,4 (overlapped), 116.1, 115.9, 62.8, 56.1, 46.5, 46.0, 38.2, 38.0, 34.3, 29.4, 22.1, 21.0 ppm. IR (KBr): 3438, 3259, 2926, 1736, 1603, 1490, 1327, 1157, 1096, 825, 702, 631 cm^−1^. HRMS (ESI-TOF) m/z: found [M + H]^+^ 479.2162; calc. for [C_28_H_31_FN_2_O_2_S]^+^: 479.2169. 17


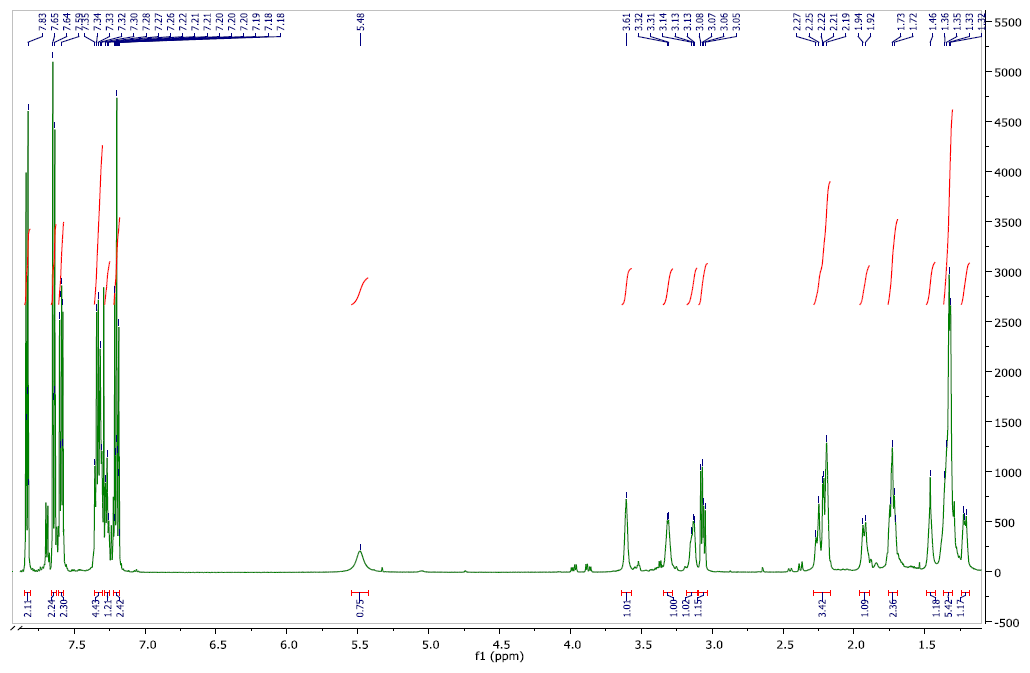


**Figure S15.** 1H NMR spectrum of 4’-fluoro-N-(((1S,4S,5R)-2-((S)-1-phenylethyl)-2- azabicyclo[3.2.1]octan-4-yl)methyl)-[1,1’-biphenyl]-4-sulfonamide


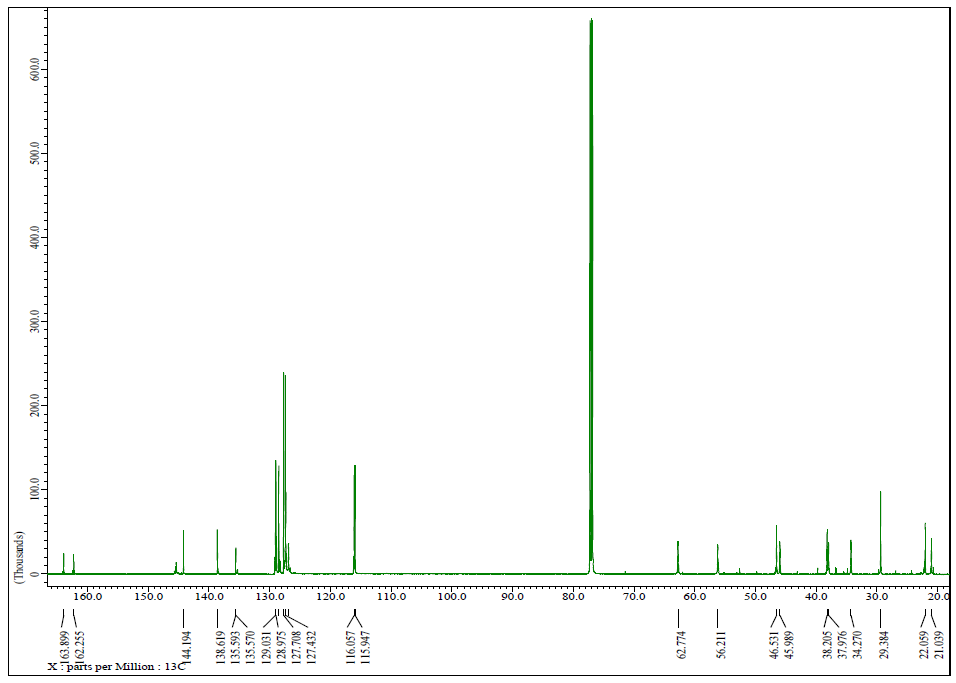


**Figure S16.** 13C NMR spectrum of 4’-fluoro-N-(((1S,4S,5R)-2-((S)-1-phenylethyl)-2- azabicyclo[3.2.1]octan-4-yl)methyl)-[1,1’-biphenyl]-4-sulfonamide 18.

4. ‘-fluoro-N-(((1S,3R,4R)-2-((S)-1-phenylethyl)-2-azabicyclo[2.2.1]heptan-3-yl)methyl)-N-(((1S,4R)-2-((S)-1-phenylethyl)-2-azabicyclo[2.2.1]heptan-3-yl)methyl)-[1,1’-biphenyl]-4-sulfonamide (13i)

Cream-coloured solid; 261 mg; 77% yield; mp. 96–98 °C; [α]D20 = +76.5 (*c* 0.8; CH_2_Cl_2_). 1H NMR (CDCl_3_, 400 MHz): δ 7.54–7.53 (m, 3H, ArH), 7.40–7.37 (m, 2H, ArH), 7.29–7.26 (m, 3H, ArH), 7.22–7.17 (m, 8H, ArH), 6.98–6.96 (m, 2H, ArH), 3.56 (s, 2H), 3.46–3.42 (q, *J* = 6.4 Hz, 2H), 2.30–2.29 (m, 2H), 2.20–2.16 (dd, *J =* 7.2, J = 3.6 Hz), 2.00–1.84 (m, 4H), 1.69–1.67 (m, 2H), 1.64–1.56 (m, 2H), 1.38–1.29 (m, 2H), 1.27–1.16 (m, 11H) ppm. 13C NMR (CDCl_3_, 125 MHz): δ 163.9, 162.2, 146.6, 143.4, 135.8 (d, *J* = 12.0 Hz), 135.5, 128.9 (d, *J* = 30.0 Hz), 128.4, 128.1, 127.9, 127.0–126.8 (d, *J* = 84.0 Hz), 116.1–116.0 (d, *J* = 21.6 Hz), 67.0, 61.1, 59.2, 53.3, 38.9, 35.1, 28.4, 23.4, 22.6 ppm. IR (KBr): 3435, 2970, 2871, 1603, 1519, 1343, 1166, 1092, 823, 702, 630 cm^−1^. HRMS (ESI-TOF) m/z: found [M + H]^+^ 678.3804 ; calc. for [C_42_H_48_FN_3_O_2_S]^+^: 678.3435.


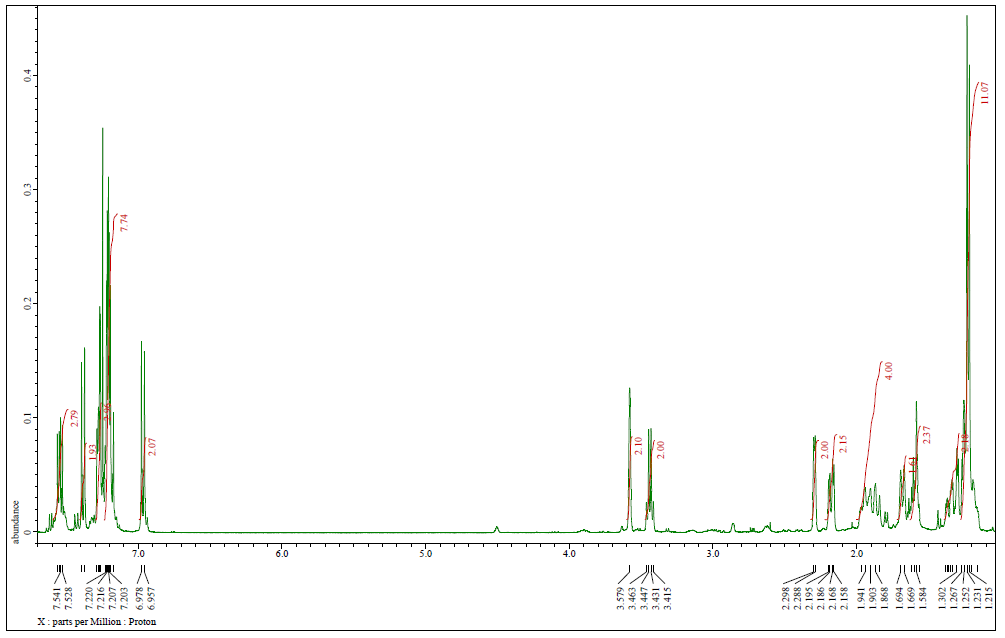


**Figure S17.** 1H NMR spectrum of 4’-fluoro-N-(((1S,3R,4R)-2-((S)-1-phenylethyl)-2-azabicyclo[2.2.1]heptan-3-yl)methyl)-N-(((1S,4R)-2-((S)-1-phenylethyl)-2-azabicyclo[2.2.1]heptan-3-yl)methyl)-[1,1’-biphenyl]-4-sulfonamide 19.


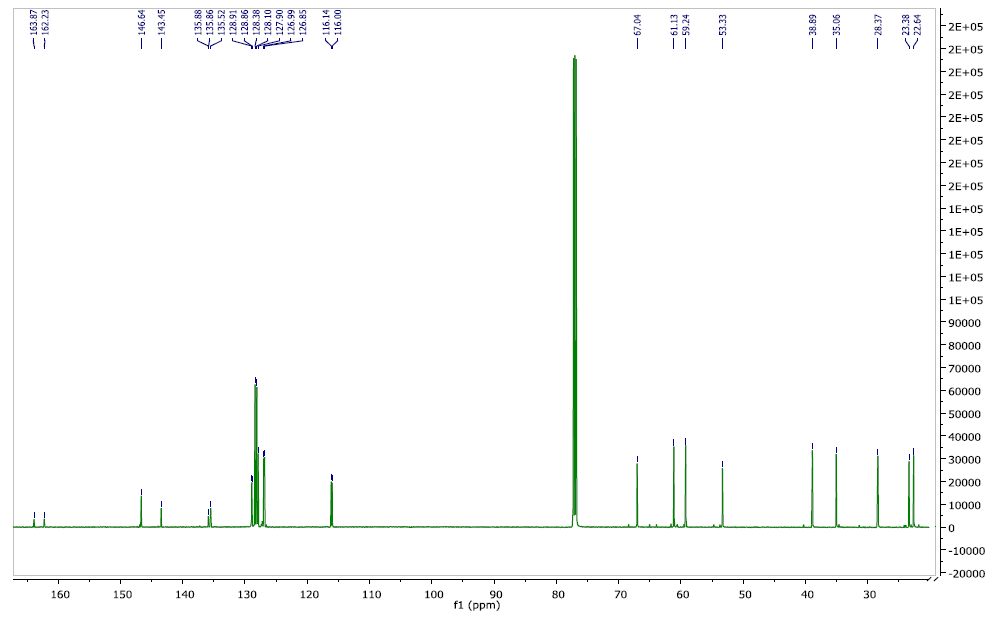


**Figure S18.** 13C NMR spectrum of 4’-fluoro-N-(((1S,3R,4R)-2-((S)-1-phenylethyl)-2-azabicyclo[2.2.1]heptan-3-yl)methyl)-N-(((1S,4R)-2-((S)-1-phenylethyl)-2-azabicyclo[2.2.1]heptan-3-yl)methyl)-[1,1’-biphenyl]-4-sulfonamide.

X-Ray Experimental Details

The compounds **9i** and **10i** crystallize in enantiomorphic, non-centrosymmetric *P*21 and *I*2 space groups, respectively. In both crystal structures, two molecules of the respective sulfonamide occur in the independent part of the unit cell and they are connected to each other by N–H···O (**9i**) and N–H···N (**10i**) hydrogen bonds. Both rings occur around the non-crystallographic inversion center. The biphenyl groups in the dimer are placed on the opposite sites of the hydrogen bonding ring and they are roughly related to each other by inversion, but the presence of 2-azanorbornane skeleton and phenylethyl group brakes this symmetry element. Such molecular assembly is responsible for suggestion of the centrosymmetric *I*2/*c* space group for **10i** by the PLATON program [4, 5]. However, this space group is excluded, as a set of (h0l) reflections violating the l = 2n rule for the presence of *c* glide plane are observed in the diffraction pattern. Therefore, the space group *I*2 is chosen. It is worth noticing that 20 aforementioned molecular assembly would also occur if the crystal structure was centrosymmetric. Thus, enantiomeric mixture of the studied compounds may supposedly crystallize in the same space group and/or very similar molecular packing as pure enantiomer.

**Table S1.** Experimental details for **9i** and **10i**.

|  | **9i** | **10i** |
| --- | --- | --- |
| Chemical formula | C_27_H_29_FN_2_O_2_S[+solvent] | C_19_H_21_FN_2_O_2_S |
| *Mr* | 464.58 | 360.44 |
| Crystal system, space group | Monoclinic, *P21* | Monoclinic, *I*2 |
| Temperature (K) | 100 | 295 |
| *a, b, c (Å)* | 9.8261 (2), 11.35426 (16), 22.1811 (5) | 21.1016 (16), 5.8230 (2), 30.4387 (19) |
| β (°) | 96.959 (2) | 108.877 (8) |
| *V (Å3)* | 2456.46 (9) | 3539.0 (4) |
| *Z* | 4 | 8 |
| μ (mm^−1^) | 0.17 | 0.21 |
| Crystal size (mm) | 0.55 × 0.48 × 0.07 | 0.44 × 0.05 × 0.04 |
| Tmin, Tmax | 0.928, 0.989 | 0.954, 0.993 |
| No. of measured, independent and observed [*I* > 2σ(*I*)] reflections | 67582, 11737, 10612 | 13986, 7487, 5611 |
| *Rint* | 0.038 | 0.024 |
| (sin θ/λ)max (Å^−1^) | 0.667 | 0.633 |
| *R*[*F*2 > 2σ(*F*2)], *wR*(*F*2), *S* | 0.038, 0.090, 1.03 | 0.048, 0.106, 1.02 |
| No. of reflections | 11737 | 7487 |
| No. of parameters | 604 | 451 |
| Δ〉_max_, Δ〉_min_ (e Å^−3^) | 0.41, −0.28 | 0.28, −0.34 |
| Absolute structure [6] | Flack x determined using 4477 quotients [(I+)−(I−)]/[(I+) + (I−)] | Flack x determined using 1967 quotients [(I+)−(I−)]/[(I+) + (I−)] |
| Absolute structure parameter | −0.004 (16) | −0.03 (5) |

CCDC 2027433–2027434 contain the supplementary crystallographic data for this paper. The data can be obtained free of charge from The Cambridge Crystallographic Data Centre via [www.ccdc.cam.ac.uk/structures](http://www.ccdc.cam.ac.uk/structures).

**Table S2.** Selected geometric parameters (Å, °) for 9i and 10i.

| **9i** | | | | |
| --- | --- | --- | --- | --- |
| S1—O2 | 1.4364 (19) | S2—O3 | 1.429 (2) | |
| S1—O1 | 1.4441 (18) | S2—O4 | 1.4377 (19) | |
| S1—N2 | 1.611 (2) | S2—N4 | 1.621 (2) | |
| S1—C16 | 1.775 (3) | S2—C43 | 1.768 (2) | |
| F1—C25 | 1.364 (3) | F2—C52 | 1.359 (3) | |
| N1—C1 | 1.475 (3) | N3—C33 | 1.474 (4) | |
| N1—C6 | 1.481 (3) | N3—C28 | 1.480 (3) | |
| N1—C8 | 1.485 (3) | N3—C35 | 1.484 (4) | |
| N2—C2 | 1.482 (3) | N4—C29 | 1.473 (3) | |
| C1—C2 | 1.529 (3) | C28—C29 | 1.528 (4) | |
| C2—C3 | 1.535 (3) | C29—C30 | 1.540 (4) | |
| C3—C7 | 1.530 (3) | C30—C34 | 1.529 (4) | |
| C3—C4 | 1.551 (3) | C30—C31 | 1.548 (4) | |
| C4—C5 | 1.556 (3) | C31—C32 | 1.556 (4) | |
| C5—C6 | 1.545 (3) | C32—C33 | 1.555 (4) | |
| C6—C7 | 1.530 (3) | C33—C34 | 1.524 (4) | |
| C8—C10 | 1.518 (3) | C35—C37 | 1.523 (4) | |
| C8—C9 | 1.534 (3) | C35—C36 | 1.535 (4) | |
| C10—C15 | 1.391 (3) | C37—C42 | 1.384 (4) | |
| C10—C11 | 1.395 (3) | C37—C38 | 1.398 (4) | |
| C11—C12 | 1.384 (4) | C38—C39 | 1.384 (4) | |
| C12—C13 | 1.394 (4) | C39—C40 | 1.387 (5) | |
| C13—C14 | 1.378 (4) | C40—C41 | 1.383 (5) | |
| C14—C15 | 1.391 (4) | C41—C42 | 1.389 (5) | |
| C16—C21 | 1.384 (4) | C43—C44 | 1.382 (4) | |
| C16—C17 | 1.388 (4) | C43—C48 | 1.393 (3) | |
| C17—C18 | 1.391 (4) | C44—C45 | 1.383 (3) | |
| C18—C19 | 1.399 (4) | C45—C46 | 1.399 (3) | |
| C19—C20 | 1.397 (4) | C46—C47 | 1.393 (3) | |
| C19—C22 | 1.493 (3) | C46—C49 | 1.485 (3) | |
| C20—C21 | 1.381 (4) | C47—C48 | 1.391 (3) | |
| C22—C27 | 1.394 (4) | C49—C50 | 1.393 (4) | |
| C22—C23 | 1.398 (4) | C49—C54 | 1.401 (3) | |
| C23—C24 | 1.391 (4) | C50—C51 | 1.386 (4) | |
| C24—C25 | 1.374 (4) | C51—C52 | 1.374 (4) | |
| C25—C26 | 1.377 (4) | C52—C53 | 1.370 (4) | |
| C26—C27 | 1.389 (4) | C53—C54 | 1.394 (3) | |
| O2—S1—O1 | 118.96 (11) | O3—S2—O4 | 120.23 (11) | |
| O2—S1—N2 | 108.01 (11) | O3—S2—N4 | 109.01 (12) | |
| O1—S1—N2 | 105.65 (11) | O4—S2—N4 | 104.64 (11) | |
| O2—S1—C16 | 108.17 (11) | O3—S2—C43 | 107.55 (12) | |
| O1—S1—C16 | 106.76 (11) | O4—S2—C43 | 107.35 (12) | |
| N2—S1—C16 | 108.97 (11) | N4—S2—C43 | 107.45 (12) | |
| C1—N1—C6 | 110.79 (18) | C33—N3—C28 | 110.9 (2) | |
| C1—N1—C8 | 109.01 (18) | C33—N3—C35 | 116.2 (2) | |
| C6—N1—C8 | 114.05 (18) | C28—N3—C35 | 107.9 (2) | |
| C2—N2—S1 | 121.87 (16) | C29—N4—S2 | 120.39 (19) | |
| N1—C1—C2 | 111.38 (19) | N3—C28—C29 | 113.2 (2) | |
| N2—C2—C1 | 110.90 (19) | N4—C29—C28 | 110.3 (2) | |
| N2—C2—C3 | 109.66 (19) | N4—C29—C30 | 112.3 (2) | |
| C1—C2—C3 | 110.59 (19) | C28—C29—C30 | 110.8 (2) | |
| C7—C3—C2 | 107.85 (19) | C34—C30—C29 | 109.2 (2) | |
| C7—C3—C4 | 102.2 (2) | C34—C30—C31 | 101.8 (2) | |
| C2—C3—C4 | 110.35 (19) | C29—C30—C31 | 110.1 (2) | |
| C3—C4—C5 | 104.98 (19) | C30—C31—C32 | 105.2 (2) | |
| C6—C5—C4 | 105.01 (19) | C33—C32—C31 | 104.7 (2) | |
| N1—C6—C7 | 108.07 (19) | N3—C33—C34 | 107.5 (2) | |
| N1—C6—C5 | 114.22 (19) | N3—C33—C32 | 115.0 (2) | |
| C7—C6—C5 | 102.10 (19) | C34—C33—C32 | 102.2 (2) | |
| C6—C7—C3 | 100.77 (19) | C33—C34—C30 | 101.1 (2) | |
| N1—C8—C10 | 109.37 (19) | N3—C35—C37 | 107.7 (2) | |
| N1—C8—C9 | 111.41 (19) | N3—C35—C36 | 113.1 (2) | |
| C10—C8—C9 | 109.2 (2) | C37—C35—C36 | 110.8 (2) | |
| C15—C10—C8 | 121.2 (2) | C42—C37—C35 | 121.7 (3) | |
| C11—C10—C8 | 120.2 (2) | C38—C37—C35 | 119.5 (3) | |
| **10i** | | | | |
| S1—O2 | 1.431 (3) | S2—O3 | | 1.436 (3) |
| S1—O1 | 1.437 (3) | S2—O4 | | 1.436 (3) |
| S1—N2 | 1.622 (3) | S2—N4 | | 1.602 (3) |
| S1—C8 | 1.781 (4) | S2—C27 | | 1.775 (4) |
| F1—C17 | 1.361 (5) | F2—C36 | | 1.365 (6) |
| N1—C5 | 1.498 (5) | N3—C24 | | 1.494 (5) |
| N1—C1 | 1.502 (4) | N3—C20 | | 1.507 (4) |
| N2—C7 | 1.477 (5) | N4—C26 | | 1.483 (5) |
| C1—C7 | 1.511 (5) | C20—C26 | | 1.521 (5) |
| C1—C2 | 1.537 (5) | C20—C21 | | 1.530 (5) |
| C2—C6 | 1.530 (6) | C21—C22 | | 1.526 (6) |
| C2—C3 | 1.538 (6) | C21—C25 | | 1.539 (5) |
| C3—C4 | 1.541 (6) | C22—C23 | | 1.551 (6) |
| C4—C5 | 1.513 (6) | C23—C24 | | 1.523 (6) |
| C5—C6 | 1.529 (5) | C24—C25 | | 1.521 (6) |
| C8—C9 | 1.385 (6) | C27—C32 | | 1.382 (6) |
| C8—C13 | 1.388 (5) | C27—C28 | | 1.386 (5) |
| C9—C10 | 1.383 (5) | C28—C29 | | 1.389 (6) |
| C10—C11 | 1.397 (5) | C29—C30 | | 1.395 (6) |
| C11—C12 | 1.388 (6) | C30—C31 | | 1.403 (6) |
| C11—C14 | 1.486 (6) | C30—C33 | | 1.487 (6) |
| C12—C13 | 1.395 (6) | C31—C32 | | 1.383 (6) |
| C14—C19 | 1.392 (5) | C33—C38 | | 1.382 (6) |
| C14—C15 | 1.398 (6) | C33—C34 | | 1.385 (6) |
| C15—C16 | 1.377 (6) | C34—C35 | | 1.389 (7) |
| C16—C17 | 1.363 (6) | C35—C36 | | 1.358 (7) |
| C17—C18 | 1.368 (7) | C36—C37 | | 1.357 (7) |
| C18—C19 | 1.385 (6) | C37—C38 | | 1.378 (7) |
| O2—S1—O1 | 118.48 (19) | O3—S2—O4 | | 118.6 (2) |
| O2—S1—N2 | 109.03 (18) | O3—S2—N4 | | 109.02 (18) |
| O1—S1—N2 | 107.15 (18) | O4—S2—N4 | | 107.43 (18) |
| O2—S1—C8 | 107.08 (19) | O3—S2—C27 | | 106.64 (19) |
| O1—S1—C8 | 107.97 (18) | O4—S2—C27 | | 108.1 (2) |
| N2—S1—C8 | 106.56 (19) | N4—S2—C27 | | 106.47 (19) |
| C5—N1—C1 | 104.8 (3) | C24—N3—C20 | | 103.3 (3) |
| C7—N2—S1 | 115.7 (3) | C26—N4—S2 | | 117.9 (3) |
| N1—C1—C7 | 114.0 (3) | N3—C20—C26 | | 111.7 (3) |
| N1—C1—C2 | 102.2 (3) | N3—C20—C21 | | 103.6 (3) |
| C7—C1—C2 | 112.5 (3) | C26—C20—C21 | | 113.6 (3) |
| C6—C2—C1 | 101.7 (3) | C22—C21—C20 | | 108.5 (3) |
| C6—C2—C3 | 102.1 (3) | C22—C21—C25 | | 101.2 (3) |
| C1—C2—C3 | 106.8 (3) | C20—C21—C25 | | 101.2 (3) |
| C2—C3—C4 | 103.0 (3) | C21—C22—C23 | | 102.3 (3) |
| C5—C4—C3 | 102.3 (3) | C24—C23—C22 | | 102.9 (3) |
| N1—C5—C4 | 107.6 (3) | N3—C24—C25 | | 100.6 (3) |
| N1—C5—C6 | 104.0 (3) | N3—C24—C23 | | 111.2 (4) |
| C4—C5—C6 | 101.4 (3) | C25—C24—C23 | | 102.6 (3) |
| C5—C6—C2 | 92.7 (3) | C24—C25—C21 | | 93.0 (3) |
| N2—C7—C1 | 111.9 (3) | N4—C26—C20 | | 111.9 (3) |
| C9—C8—S1 | 118.9 (3) | C32—C27—S2 | | 119.6 (3) |
| C13—C8—S1 | 120.8 (3) | C28—C27—S2 | | 120.8 (3) |

**Table S3.** Selected hydrogen-bond parameters for 9i and 10i.

| ***D—H···A*** | ***D—H (Å)*** | **H···*A* (Å)** | ***D*···*A* (Å)** | ***D*—H···*A* (°)** |
| --- | --- | --- | --- | --- |
| **9i** | | | | |
| N2—H2N···O4i | 0.78 (3) | 2.29 (3) | 3.039 (3) | 161 (3) |
| C2—H2···F2ii | 0.98 | 2.47 | 3.150 (3) | 126.0 |
| C7—H7A···F2iii | 0.97 | 2.58 | 3.535 (3) | 169.4 |
| C21—H21···O4i | 0.93 | 2.49 | 3.390 (3) | 162.9 |
| N4—H4N···O1iv | 0.85 (3) | 2.16 (3) | 3.001 (3) | 170 (3) |
| C34—H34B···O1iv | 0.97 | 2.59 | 3.543 (3) | 167.9 |
| **10i** | | | | |
| N1—H1N···O4i | 1.02 | 2.61 | 3.288 (4) | 123.8 |
| N2—H2N···N3 | 1.09 | 2.00 | 3.077 (5) | 167.8 |
| C4—H4A···O4 | 0.97 | 2.56 | 3.376 (5) | 141.6 |
| C18—H18···O2v | 0.93 | 2.44 | 3.337 (6) | 163.1 |
| N3—H3N···O1iv | 1.00 | 2.17 | 3.153 (5) | 169.0 |
| N4—H4N···N1 | 0.90 | 2.02 | 2.895 (5) | 161.7 |
| C35—H35···O3vi | 0.93 | 2.37 | 3.229 (6) | 153.4 |

References

1. Kamińska, K.; Wojaczyńska, E.; Wietrzyk, J.; Turlej, E.; Błażejczyk, A.; Wieczorek, R. Synthesis, structure and antiproliferative activity of chiral polyamines based on a 2-azanorbornane skeleton. *Tetrahedron: Asymmetry* **2016**, *27*, 753–758, doi:10.1016/j.tetasy.2016.06.009.
2. Jin, H.; Lazerwith, S.E.; Martin, T.A.T.; Bacon, E.M.; Cottell, J.J.; Cai, Z.R.; Pyun, H.; Morganelli, P.A.; Ji, M.; Taylor, J.G.; Chen, et al. Polycyclic-Carbamoylpyridone Compounds And Their Pharmaceutical Use. WO 2014/100323 Al. 26 June 2020.
3. Samadaei, M.; Pinter, M.; Senfter, D.; Madlener, S.; Rohr-Udilova, N.; Iwan, D.; Kamińska, K.; Wojaczyńska, E.; Wojaczyński, J.; Kochel, A. Synthesis and Cytotoxic Activity of Chiral Sulfonamides Based on the 2-Azabicycloalkane Skeleton. *Mol.* **2020**, *25*, 2355, doi:10.3390/molecules25102355.
4. Spek, A.L. What makes a crystal structure report valid?. *Inorganica Chim. Acta* **2018**, *470*, 232–237, doi:10.1016/j.ica.2017.04.036.
5. Spek, A.L. checkCIF validation ALERTS: what they mean and how to respond. *Acta Crystallogr. Sect. E Crystallogr. Commun.* **2020**, *76*, 1–11, doi:10.1107/s2056989019016244.
6. Parsons, S.; Flack, H.D.; Wagner, T. Use of intensity quotients and differences in absolute structure refinement. *Acta Crystallogr. Sect. B Struct. Sci. Cryst. Eng. Mater.* **2013**, *69*, 249–259, doi:10.1107/s2052519213010014.

| 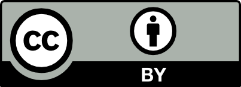 | **© 2020 by the authors. Submitted for possible open access publication under the terms and conditions of the Creative Commons Attribution (CC BY) license (http://creativecommons.org/licenses/by/4.0/).** |
| --- | --- |
